# Derivatives of 10,16-Dihydroxyhexadecanoic Acid Isolated from Tomato (*Solanum lycopersicum*) as Potential Material for Aliphatic Polyesters

**DOI:** 10.3390/molecules16064923

**Published:** 2011-06-15

**Authors:** Daniel Arrieta-Baez, Miguel Cruz-Carrillo, Mayra Beatriz Gómez-Patiño, L. Gerardo Zepeda-Vallejo

**Affiliations:** Departamento de Química Orgánica, Escuela Nacional de Ciencias Biológicas-IPN, Prolongación de Carpio y Plan de Ayala S/N, Colonia Santo Tomás, D.F. 11340, Mexico

**Keywords:** cutin, tomato, 16-hydroxyacids, dimer, trimer

## Abstract

The main monomer of tomato cuticle, 10,16-dihydroxyhexadecanoic acid (or 10,16-dihydroxypalmitic acid; 10,16-DHPA), was isolated and used to efficiently synthesize two different monomers (16-hydroxy-10-oxo-hexadecanoic and 7-oxohexa-decanedioic acids) in addition to a dimer and linear and branched trimers. These compounds were fully characterized using NMR and MS techniques and could be used as starting materials for the synthesis of a wide range of chemicals and bio-polyesters, particularly the latter due to their physical properties, non-toxicity, and relative abundance among raw materials.

## 1. Introduction

There is a great need to develop chemistry that is based on the use of biodegradable and renewable resources [1]. Natural aliphatic polyesters are amongst the most important biocompatible and biodegradable materials that have received much recent attention; their applications in particular fields, such as agriculture, packaging, fiber, and biomedical research (e.g., tissue engineering, surgical suture, gene therapy, and controlled drug delivery) have grown significantly due to their availability as novel products with better performance characteristics [2,3]. Normally, polyester synthesis is performed by ester interchange reactions or by direct esterification of hydroxyacids or diacid/diol combinations from agro-resources, chemical synthesis and fossil resources (e.g., lactic acid-, fatty acid-derived materials, ε-caprolactones, different diols, adipic, sebacic or succinic acid, *etc.*) [4,5,6,7,8]. Development of these innovative biopolymer materials has been underway for a number of years, and continues to be an area of interest for many research fields [2,3,9].

Appreciable amounts of natural polyesters occur in higher plants as structural components; cutin, present in the cuticle that covers the aerial parts of plants, and suberin, which forms part of the periderm in woody plants, are both examples of natural polyesters [10,11,12]. Their monomeric composition is usually complex and differs among plant species [10,11,13,14,15,16,17], and this complexity probably explains why the use of cutin and suberin as commercial sources of “green” chemicals has not been well-explored. Monomers such as ω-hydroxyl-hexadecanoic acid, α,ω-hexadecanedioic acid, and α,ω-hexadecanediol, which are present in cutin and suberin, are found commercially and have been used in biological and polymeric studies [18,19,20,21]. On the other hand, aliphatic α,ω-dicarboxylic acids are used in the manufacture of engineered plastics, perfumes, lubricants, and adhesives [7].

Tomato cutin is one of the most studied cuticles [11,15,22,23]. Various depolymerization protocols have indicated that the major monomers in this unique natural polyester are 10,16-dihydroxy-hexadecanoic acid (10,16-dihydroxypalmitic acid, 10,16-DHPA; more than 70%) and 16-hydroxy-hexadecanoic acid [15,23,24]. However, studies describing the chemical or enzymatic reactions with purified monomers isolated from tomato cutin, have not yet been explored. These reactions require a robust chemical analysis in order to know the use of the monomers for the industrial applications.

Thus, the present work describes the use of 10,16-DHPA to efficiently synthesize the 16-dihydroxy-10-oxo-hexadecanoic acid (a monomer present in lime cuticle) [13,16,25], 7-oxohexadecanendioic acid [26], as well as a dimer and linear and branched trimer compounds. These products were characterized by nuclear magnetic resonance (NMR) and mass spectrometry (MS) and they could be used as starting materials in the preparation of different aliphatic polyesteres with potential industrial applications.

## 2. Results and Discussion

### 2.1. Isolation of 10,16-Dihydroxyhexadecanoic Acid *(**1**)*

The monomer was isolated from tomato peels using previously reported protocols [15,23]. It was characterized by NMR analysis, and confirmed using various mass spectrometry techniques. EI-MS of compound **1** showed a molecular ion at *m/z* 289 [M+H]^+^, which is consistent with the molecular formula C_16_H_32_O_4_. Dehydration fragments were observed at *m/z* 271 and 253 ([M+H−H_2_O]^+^ and [M+H−2H_2_O]^+^ respectively), and fragments observed at *m/z* 131, 141, and 169 suggested the hydroxyl position at C10. 10,16-Dihydroxyhexadecanoic acid has been synthesized and compared with the isolated monomer from tomato [27]. Both isomers, 9,16-DHPA and 10,16-DHPA, are usually described as mixtures in previously published studies [28,29]. In this study, we found 10,16-DHPA as the main isomer isolated from tomato cutin according to the mass analysis from different derivatives (Table 1).

**Table 1:** EI-MS data of 10,16-DHPA and its derivatives EI-MS data of 10,16-DHPA and its derivatives. 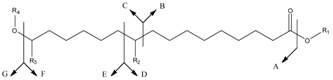

Compounds	MH^+^	A	B	C	D	E	F	G
**1**	289	253(10)	157(14) 140(−H_2_O)(9)	131(26)	169(-H_2_O)(100)	---	271(1)	18(4)
R_1_ = H R_2_ = OH								
R_3_ = H R_4_ = H								
**2**	403	385(5)	---	245(13)	187	---	271	132(11)
R_1_ = H R_2_ = OH					169(−H_2_O)(11)		253(−H_2_O)(8)	
R_3_ = H R_4_ = TBS								
**3**	517	499(7)	---	359(9)	301(7)	---	---	132(11)
R_1_ = H R_2_ = OTBS								
R_3_ = H R_4_ = TBS								
**4**	317	271(9)	185(22)	---	215(33)	101(40)	299(45)	---
R_1_ = CH_2_CH_3_ R_2_ = OH								
R_3_ = H R_4_ = H								
**5**	300	----	157(79)	---	185(62)	----	---	----
R_1_ = H R_2_ = (=O)								
R_3_ = (=O) R_4_ = H								
**6b**	---	269(34)	---	---	185(28)	---	---	251(35) ^b^
R_1_ = H R_2_ = (=O)								
R_3_ = H R_4_ = H								

^a^ Relative peak intensities are indicated in parentheses; ^b^ [269−H2O]^+^.

### 2.2. Preparation OF 7-Oxohexadecanedioic ACID *(**5**)*

10,16-DHPA was oxidized using PCC as oxidant reagent. The reaction mixture was purified by column chromatography and eluted with hexane-AcOEt (3:7, v/v) to give 7-oxohexadecanedioic acid (**5**) as the main product. EI-MS showed a molecular ion at *m/z* 300 [M+H]^+^, which is consistent with the molecular formula C_16_H_28_O_5_. Fragments observed at *m/z* 185 and 157 confirmed the presence of the carbonyl group at position C7. The successful conversion of hydroxyl groups to keto and carboxyl groups is evident from the ^1^H-NMR spectra that show the complete disappearance of signals corresponding to 10,16-dihydroxy groups at 3.60 ppsm and the appearance of ^1^H-NMR signals for the product at 2.36 (m, 8H) and 1.62 ppm (m, 8H), respectively. The ^13^C-NMR spectra show the presence of the keto group at 211.73 ppm and two carboxylic groups at 180.23 and 179.96 ppm (HOCO-16 and HOCO-1, respectively) to confirm the complete oxidation.

Aliphatic α,ω-dicarboxylic acids (α,ω-diacids) are used for the manufacture of engineered plastics, perfumes, fragrances, lubricants and adhesives. They are mainly produced by chemical protocols from non-renewable petrochemical feedstock, and these chemical routes can be tedious and result in unwanted byproducts [7].

Some long-chain unsaturated and epoxidized dicarboxylic acids (9,10-epoxy octadecanedioic and 1,18-*cis*-9-octanedioic acids for instance) can be found in Nature [7]. These and related monomers would be useful to design and synthesize unique functional polyesters that would be also biodegradable. However, they are difficult to synthesize by chemical methods and are currently commercially unavailable.

### 2.3. Preparation OF 16-Hydroxy-10-Oxo-Hexadecanoic ACID *(**6b**)*

16-Hydroxy-10-oxo-hexadecanoic acid (**6b**) is a natural monomer identified in different cuticles [13,16,25]. In order to prepare this monomer, compound **2** was oxidized with PCC to give the respective keto group (**6a**). The protective group of HO-16 (TBS) was removed from compound **6a** with TBAF to give compound **6b** in good yield.

Compound **6a** was used to confirm the presence of the OH group at position C-10 in monomer (**1**) isolated from tomato. Its corresponding EI-MS spectrum did not yield a molecular ion. However, the observed fragments were consistent with the molecular formula C_22_H_44_O_4_Si, *m/z* 383 [400−H2O]^+^, and the fragment at *m/z* 185, which corresponds to the cleavage at C10-C11, confirms the presence of the C=O at carbon 10.

Other fragments were found in different derivatives to corroborate the hydroxyl position at C10; compound **2** produced a fragment at *m/z* 245 and compound **3** at *m/z* 301 and 359 (Table 1).

### 2.4. Preparation and Characterization OF Dimer ***7*** and Trimer ***8***

**Scheme 1:**
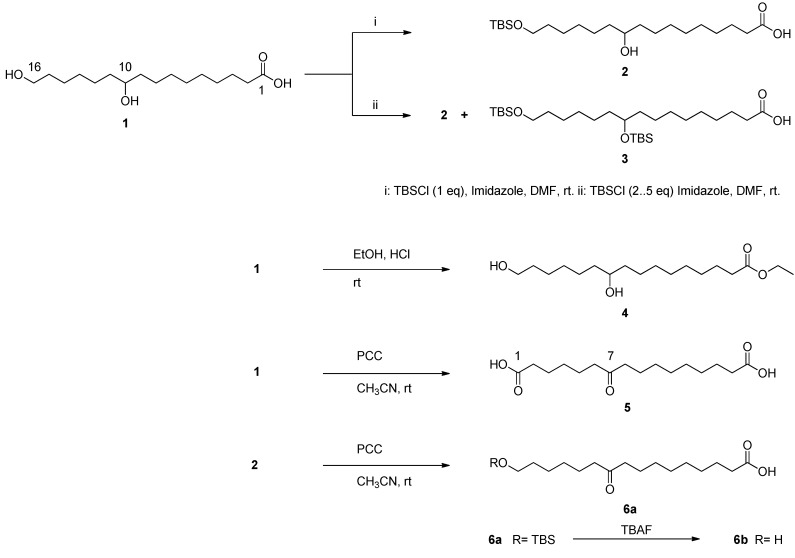
Protective and oxidative reactions of 10,16-dihydroxyhexadecanoic acid (10,16-DHPA, **1**).

**Scheme 2:**
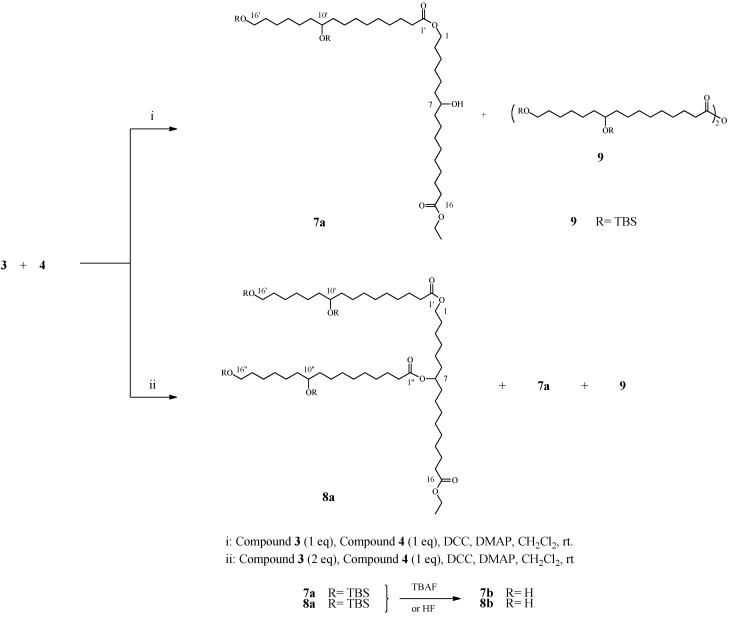
Dimer and branched trimer obtained from 10,16-DHPA derivatives (Numeration and NMR assignments were given according to IUPAC names).

Dimer and trimer 10,16-DHPA derivatives were prepared according to Scheme 1 and Scheme 2. Different equivalents of *t*-butyldimethylsilyl chloride were used in order to successfully protect either one or both hydroxyl groups of the monomer. Although the reaction yielded mainly one protected monomer, a small amount of other compounds was present within the mixture. Radial chromatography was an efficient technique to separate the mixture using CH_2_Cl_2_ and CH_2_Cl_2_-MeOH (95:5, v/v) as eluents. The reaction gave approximately 85%–90% yield of a yellow pale oils characterized by NMR as 10-hydroxy-16-(*ter*-butyl-dimethylsilyloxy) hexadecanoic acid (**2**) and 10-16-bis(*ter*-butyldimethyl-silyloxy) hexadecanoic acid (**3**).

The ^1^H-NMR spectrum of compound **2** showed a singlet at δ 0.04 ppm while compound **3** showed two singlets at δ 0.04 and 0.03 ppm, indicating the presence of 1 and 2 TBS groups, respectively. Small but significant differences in the displacement of silyl methyl groups can be found in the ^13^C spectra to assign the TBS protection of hydroxyl groups at C10 and/or C16. Compound **2** showed a peak at δ −4.41 ((CH_3_)_2_SiO-C16) while compound **3** showed two signals at δ −4.41 and −5.26 ((CH_3_)_2_SiO-C10), thus confirming the presence of TBS in both hydroxyl groups.

It is well known that dihydroxy methyl esters undergo rapid oligomerization [27], and this behavior is also present in the monomer extraction procedure. Two small fractions corresponding to the methyl 10,16-dihydroxyhexadecanoate and polymeric fractions were identified by NMR analysis. The proportions of these fragments increased when the pH was lower than 5 during the monomer extraction, indicating that the reaction between the monomer and methanol, and the subsequent oligomerization process, is due to the acidic conditions. In order to avoid this, ethyl ester of 10,16-DHPA (**4**) was prepared by treating **1** with anhydrous EtOH under a catalytic amount of HCl, thereby producing only the solid product **4**, which was fully characterized by NMR and EI-MS.

Attempts to prepare the corresponding co-dimer using equimolar amounts of compounds **3** and **4,** were successful by using DCC and DMAP in CH_2_Cl_2_. The reaction was followed by TLC (CHCl_3_-MeOH, 95:5, v/v) and was stopped when compound **4** was consumed after four days. Two products were separated by radial chromatography and characterized by NMR and MS as compound **7a** and **9**. The ^1^H-NMR of compound **7a** (R_f_ 0.7, CHCl_3_-MeOH, 98:2, v/v) showed a triplet at δ 4.05 (H-16, 2H), indicating that the ester group is linking both monomers. The ^13^C-NMR spectrum revealed two peaks at δ 173.98 and 173.87, corresponding to both carbonyl groups. The structure of the co-dimer was confirmed by HMBC through cross-peak linking H-16 at δ 4.05 with COOH at δ 173.87).

Deprotection of compound **7a** was performed with HF, and the main product was isolated and characterized as compound **7b**. HR-FABMS revealed a molecular ion at *m/z* 586.4809 (calculated 586.4809) consistent with molecular formula C_34_H_66_O_7_.

Compound **9** was characterized as 10,16-dihydroxyhexadecanoic anhydride. ^1^H-NMR analysis revealed a similar spectrum as compound **3**. However, compound **9** had a different R_f_ (0.85, CHCl_3_-MeOH, 95:5, v/v) as compared to compound **3** (R_f_ 0.56, CHCl_3_-MeOH, 95:5, v/v).

In a new reaction, 2.5 equiv. of compound **3** and 1 equiv. of compound **4** yielded a branched trimer. After five days, compound **4** was consumed, and three products were separated and purified under the conditions above described. In addition to compounds **7a** (30 mg, 11%) and **9** (12 mg, 2%), the predominant compound was **8a** (285 mg, 65%), according to NMR and MS analyses.

^1^H-NMR of compound **8a** (R_f_ 0.65, CHCl_3_-MeOH, 98:2, v/v) showed a quintuplet at δ 4.85, indicating the presence of an ester bond in position C10, as well as a triplet at δ 4.04 (H-16, 2H), demonstrating the presence of an ester group at C16, as previously shown. Upon ^13^C-NMR analysis, three peaks at δ 173.94, 173.84, and 173.66, corresponding to the three carbonyl groups, were observed. The trimer was confirmed by HMBC thorough cross-peaks of H-10 at δ 4.80 with CO at δ 173.8 and H-16 at δ 4.05 with CO at δ 173.8. The NMR data of these compounds were compared with those previously reported for isolated oligomers from the tomato peel cutin [30,31].

Deprotection of compound **8a** was undertaken with HF, and the main product was isolated and characterized as compound **8b**. HR FABMS yield a molecular ion at *m/z* 856.7003 consistent with molecular formula C_50_H_96_O_10_.

### 2.5. Preparation of Linear Trimer

**Scheme 3:**
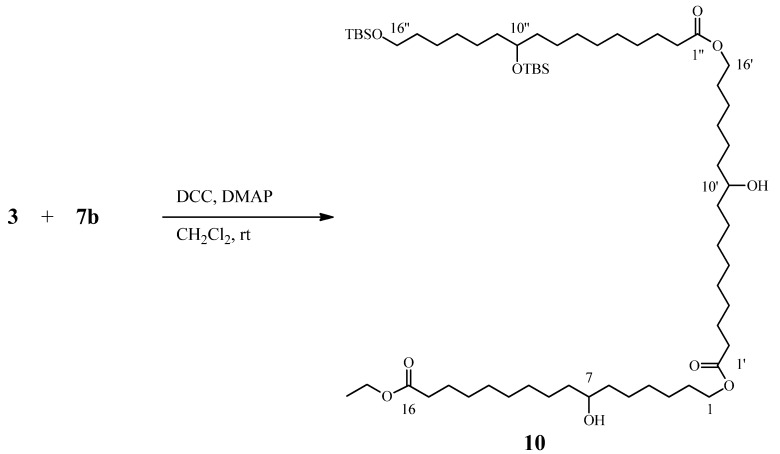
Preparation of a linear trimer from compounds **3** and **7b.**

Production of the linear trimer was attempted using equimolar amounts of compounds **3** and **6b** (Scheme 3). After 6 days, the reaction was completed. However, different products with very close R_f_’s were formed, and the linear trimer was recovered in low yield. The ^1^H-NMR spectrum showed similarities with compound **7a**: a quartet at δ 4.12, a triplet at δ 4.05, triplet at δ 2.28, and singlets at δ 0.89, 0.88, 0.05, and 0.03 (-OTBS groups). However, there was no presence of a quintuplet at δ 4.85, as in compound **8a**, indicating the esterification in the secondary OH.

## 3. Experimental

### 3.1. General

*N,N’*-Dicyclohexylcarbodiimide (DCC), 4-dimethylamino pyridine (DMAP), imidazole, *t*-butyl-dimethylsilyl chloride (TBSCl), acetonitrile, dioxane, DMF, methanol, and toluene of reagent grade were purchased from Aldrich (St. Louis, MO) and used without further purification.

NMR spectra were acquired on a Varian 500 MHz NMR system or on a Varian 300 MHz (Palo Alto, CA) as specified. The ^1^H and ^13^C chemical shifts are given in units of δ (ppm) relative to tetramethylsilane (TMS), where δ (TMS) = 0 ppm.

Mass spectral (MS) data were acquired on a JEOL JMS-GCmate II. Masses were scanned between 200 and 1500 *m/z*, using power ionization values between 60 and 250 V depending on whether molecular ions or fragments were desired. HRFAB were registered on a JEOL JMS-700 using *m*-nitrobenzyl alcohol as a matrix.

### 3.2. Isolation of 10,16-Dihydroxypalmitic Acid *(10,16-DHPA, **1**)*

10,16-DHPA was obtained by depolymerization of tomato cutin as previously described [12,15,22], and its structural characterization was determined by comparing the NMR and MS data with those previously reported [27].

### 3.3. Synthesis

#### 3.3.1. Preparation of 10-hydroxy-16-OTBS-PA (**2**) and 10,16-di-OTBS-PA (**3**)

Hydroxyl groups of 10,16-DHPA were protected using various equivalents of *t*-butyldimethylsilyl chloride (TBSCl) in separate reactions. A mixture of compound **1** (100 mg, 0.34 mmol), TBSCl (52.3 mg, 0.34 mmol), and imidazole (23.6 mg, 0.34 mmol) in DMF (2 mL) was used to prepare compound **2**. In a separate reaction, a mixture of compound **1** (100 mg, 0.34 mmol), TBSCl (104.6 mg, 0.69 mmol), and imidazole (47.2 mg, 0.69 mmol) in DMF (3 mL) was used to prepare compound **3**. Both reactions were stirred at room temperature for 1 h, then a mixture of hexane-CH_2_Cl_2_ (1:1, v:v) was added after which the mixture was filtered. The organic phase was washed with a saturated solution of ammonium chloride (1 M, 50 mL) and water (3 × 50 mL) and dried over anhydrous Na_2_SO_4_. After evaporation of the solvent at reduced pressure, both crude products were purified by radial chromatography (chromatotron). They were eluted successively with hexane, CH_2_Cl_2_, and CH_2_Cl_2_-MeOH (9:1 and 4:1; v:v), giving pure compounds **2** and **3** as colorless oils.

*16-(tert-Butyldimethylsilyloxy)-10-hydroxyhexadecanoic acid* (**2**). Colorless oil (338.4 mg, 84% yield) ^1^H-NMR (300 MHz, CDCl_3_) δ 3.60 (t and m, *J* = 6.57 Hz, 3H) CH-10 and CH_2_-16, 2.34 (t, *J* = 7.46 Hz, 2H) CH_2_-2, 1.63 (quint, *J* = 7.60 Hz, 2H) CH_2_-15, 1.51 (quint, *J* = 7.70 Hz, 2H) CH_2_-3, 1.43 (m, 4H) CH_2_-9 and CH_2_-11, 1.30 (m, 16H) CH_2_-4 to CH_2_-8 and CH_2_-12 to CH_2_-14, 0.89 (s, 9H) Si-tBu, and 0.04 (s, 6H) Si(Me)_2_. ^13^C-NMR (75 MHz, CDCl_3_) δ 178.96 (CO), 72.29 (C-10), 63.01 (C-16), 37.02 and 37.01 (C-9 and C-11), 33.98, 32.65, 29.68, 29.64, 29.33, 29.13, 29.02, 25.97, 25.26, 25.19 and 24.70 (C-2 to C-8 and C-12 to C-15), 25.93 SiC(Me)_3_, 18.15 SiC(Me)_3_ and −4.41 Si(Me)_2_. FAB-HRMS (calc. for C_22_H_46_O_4_Si: 402.3165), found 402.3164. EI-MS *m/z*: 385 [M−H_2_O]^+^, 326 s[M−C_2_H_3_O_2_−OH]^+^, 245 [M−C_9_H_17_O_2_]^+^, 133 [C_6_H_16_Osi+H]^+^.

*10,16-Bis-(tert-butyldimethylsilyloxy)hexadecanoic acid* (**3**). Colorless oil (475.5 mg, 92% yield). ^1^H-NMR (300 MHz, CDCl_3_) δ 3.60 (t and m, *J* = 6.61 Hz, 3H) CH-10 and CH_2_-16, 2.33 (t, *J* = 7.33 Hz, 2H) CH_2_-2, 1.62 (quint, *J* = 6.60 Hz, 2H) CH_2_-15, 1.50 (quint, *J* = 6.60 Hz, 2H) CH_2_-3, 1.20–1.44 (m, 20H) CH_2_-4 to CH_2_-9, CH_2_-11 to CH_2_-14, 0.89 (s, 9H) Si-tBu, 0.88 (s, 9H) Si-tBu, 0.04 (s, 6H) Si(Me)_2_ and 0.03 (s, 6H) Si(Me)_2_. ^13^C-NMR (75 MHz, CDCl_3_) δ 178.90 (CO), 72.33 (C-10), 63.33 (C-16), 37.08 (C-9 and C-11), 32.82 (C-2 and C-15), 29.79, 29.66, 29.45, 29.23, 29.08, 25.81, 25.33, 25.27 and 24.73 (C-3 to C-8 and C-12 to C-14), 25.98 SiC(Me)_3_-16, 18.38 SiC(Me)_3_−16 and −5.26 Si(Me)_2_−16; 25.93 SiC(Me)_3_−10, 18.16 SiC(Me)_3_−10 and −4.41 Si(Me)_2_−10. FAB-HRMS (calc. for C_28_H_60_O_4_Si_2_: 516.4030), found 516.4030. EI-MS *m/z*: 499 [M−OH]^+^, 459 [M−C_2_H_3_O_2_]^+^, 359 [M−C_9_H_17_O_2_]^+^, 327 [459−C6H15OSi]^+^, 301 [M−C_12_H_27_OSi]^+^.

#### 3.3.2. Preparation of ethyl-10,16-dihydroxy hexadecanoate (**4**)

HCl (0.05 mL) was added to a stirred solution of compound **1** (0.86 mM in absolute ethanol, 0.17 mmol). The mixture was allowed to react overnight at room temperature, and then evaporated at reduced pressure. The remaining HCl was co-evaporated with ethanol, and product **4** was recovered without further purification from CH_2_Cl_2_.

*Ethyl 10,16-dihydroxyhexadecanoate* (**4**). Pale yellow wax (300.5 mg, 95% yield). ^1^H-NMR (300 MHz, CDCl_3_) δ 4.11 (q, *J* = 7.14 Hz, 2H) –OCH_2_CH_3_, 3.63 (t and m, *J* = 9.1 Hz, 3H) CH-10 and CH_2_-16, 2.27 (t, *J* = 7.52 Hz, 2H) CH_2_-2, 1.60-1.22 (m, 27H) –OCH_2_CH_3_, CH_2_-3 to CH_2_-9 and CH_2_-11 to CH_2_-15. ^13^C-NMR (75 MHz, CDCl_3_) δ 173.92 (CO), 71.94 (C-10), 62.99 (C-16), 37.47 (C-9), 37.35 (C-11), 34.37 (C-2), 32.71 (C-15), 29.40, 29.25, 29.21, 29.19, 29.08, 28.91, 25.53, 25.40 and 24.76 (C-3 to C-8 and C-12 to C-14), 60.16 (–OCH_2_CH_3_), 14.24 (–OCH_2_CH_3_). EI-MS *m/z*: 317 [M+H]^+^, 299 [M−H_2_O]^+^, 253 [299−C2H5O]^+^, 235 [253−H2O]^+^, 215 [M−C_6_H_13_O]^+^, 185 [M−C_7_H_15_O_2_]^+^, 101 [M−C_12_H_23_O_3_]^+^.

#### 3.3.3. Oxidation of 10,16-DHPA (**1**) and compound **2** with pyridinium chlorochromate (PCC)

To a stirred solution of PCC (0.23 mmol) in CH_3_CN, 10,16-DHPA (Compound **1**) (0.12 mmol) or compound **2** (0.12 mmol) dissolved in CH_3_CN was added in separated reactions. After 24 h, the reaction was stopped and filtered on celite. The solvent was evaporated under vacuum, and the residue was redissolved in ethyl ether, washed with brine, dried, and concentrated to give mainly compounds **5** and **6a**.

*7-Oxohexadecanedioic acid* (**5**). Brown solid, (32 mg, 63%). ^1^H-NMR (300 MHz, CDCl_3_) δ 2.36 (m, 8H) CH_2_-2,6,8 and CH_2_-15, 1.62 (m, 8H) CH_2_-3,5,9 and CH_2_-14, 1.30 (m, 10H) CH_2_-4,10,11,12 and CH_2_-13. ^13^C-NMR (75 MHz, CDCl_3_) δ 211.73 (CO-7), 180.23 (COOH-16), 179.96 (COOH-1), 42.94 (C-8), 42.52 (C-6), 34.16 (C-2), 33.96 (C-15), 29.21, 29.09, 28.90, 28.67, 24.72, 24.53, 24.37, 23.91 and 23.49 (C-3 to C-5 and C-9 to C-14) EI-HRMS *m/z* 300.1944 (C_16_H_28_O_5_, 300.1937). EI-MS *m/z* 300 [M]^+^, 241 [M−C_2_H_3_O_2_]^+^, 185 [M−C_6_H_11_O_2_]^+^, 157 [M−C_7_H_11_O_3_]^+^.

*16-(tert-Butyldimethylsilyloxy)-10-oxo-hexadecanoic acid* (**6a**). Pale yellow oil, (71 mg, 72%), ^1^H-NMR (300 MHz, CDCl_3_) δ 3.60 (t, *J* = 6.57 Hz, 2H) CH_2_-16, 2.39–2.28 (m, 6H) CH_2_-2, CH_2_-9 and CH_2_-11, 1.63-1.66 (m, 20H) CH_2_-3 to CH_2_-8 and CH_2_-12 to CH_2_-15, 0.89 (s, 9H) Si-tBu and 0.05 (s, 6H) Si(Me)_2_. ^13^C-NMR (75 MHz, CDCl_3_) δ 212.10 (CO-10), 179.40 (CO), 63.29 (C-16), 42.56 (C-9), 42.49 (C-11), 33.80 (C-2), 33.70 (C-15), 29.28, 29.14, 28.90, 28.72, 25.39, 24.71 and 24.07 (C-3 to C-8 and C-12 to C-14), 25.97 SiC(Me)_3_, 18.38 SiC(Me)_3_ and −4.41 Si(Me)_2_. EI-HRMS *m/z* 400.3008 (C_22_H_44_O_4_Si, 400.3008). EI-MS *m/z* 400 [M]^+^, 382 [M−OH]^+^, 215 [M−C_10_H_17_O_3_]^+^, 185 [M−C_12_H_27_OSi]^+^.

Deprotection of compound **6a***.* Compound **6a** (1.23 mmol) in 5 mL THF at 0 °C was treated with TBAF (5 mL, 1 M in THF, 5 mmol) and then stirred at room temperature for 2 h. The mixture was then quenched with aqueous NH_4_Cl, extracted with Et_2_O, washed with brine, and dried over anhydrous Na_2_SO_4_. The product was purified by column chromatography using CH_2_Cl_2_-MeOH (98:2, v/v) as eluent to afford compound **6b** as a wax yellow pale (31 mg, 86% yield).

*16-Hydroxy-10-oxo-hexadecanoic acid* (**6b**). Pale yellow wax (31 mg, 86%) ^1^H-NMR (300 MHz, CDCl_3_) δ 3.64 (t, *J* = 6.60 Hz, 2H) CH_2_-16, 2.38–2.33 (m, 6H) CH_2_-2, CH_2_-9 and CH_2_-11, 1.62–1.53 (m, 8H) CH_2_-3, CH_2_-8, CH_2_-12 and CH_2_-15, 1.33-1.25 (m, 12H) CH_2_-4 to CH_2_-7, CH_2_-13 and CH_2_-14. ^13^C-NMR (75 MHz, CDCl_3_) δ 214.19 (CO-10), 181.36 (CO), 64.89 (C-16), 43.56 (C-9), 42.49 (C-11), 33.80 (C-2), 33.70 (C-15), 29.28, 29.14, 28.90, 28.72, 25.39, 24.71 and 24.07 (C-3 to C-8 and C-12 to C-14). EI-MS *m/z* 269 [M−OH]^+^, 251 [M−2H_2_O]^+^, 185 [M−C_6_H_13_O]^+^.

#### 3.3.4. Dimerization of 10,16-DHPA derivatives

To a stirred solution of **3** (174 mg, 0.33 mmol) and **4** (106.3 mg, 0.33 mmol) in dry CH_2_Cl_2_ (4 mL), DMAP (41.05 mg, 0.33 mmol) and DCC (138.6 mg, 0.67 mmol) were added. The reaction mixture was then stirred at room temperature for four days. A mixture of hexane-CH_2_Cl_2_ (1:1, v:v) was added, and the reaction mixture was filtered. The filtrate was washed with saturated NaHCO_3_ and brine, and dried with anhydrous Na_2_SO_4_ after which the solvent was evaporated to dryness. The crude product was purified by radial chromatography (chromatotron) through successive elutions with hexane, CH_2_Cl_2_, and CH_2_Cl_2_-MeOH (9:1 and 4:1; v:v).

*16-Ethoxy-7-hydroxy-16-oxohexadecyl 10’,16’-bis(tert-butyldimethylsilyloxy) hexadecanoate* (**7a**). ^1^H-NMR (500 MHz, CDCl_3_) δ 4.12 (q, *J* = 7.14 Hz, 2H) –OCH_2_CH_3_, 4.05 (t, *J* = 6.72 Hz, 2H) CH_2_-1, 3.60 (m, *J* = 6.55 Hz, 4H) CH_2_-16’, CH-10’ and CH-7, 2.28 (t, *J* = 7.54 Hz, 4H) CH_2_-2’ and CH_2_-15, 1.61 (m, 4H) CH_2_-15’ and CH_2_-2, 1.50 (m, 4H) CH_2_-3’ and CH_2_-14, 1.46–1.27 (m, 44H), 1.25 (t, *J* = 7.14 Hz, 3H) –OCH_2_CH_3_, 0.89 (s, 9H) Si-tBu, 0.88 (s, 9H) Si-tBu, 0.05 (s, 6H) SiMe_2_ and 0.03 (s, 6H) SiMe_2_. ^13^C-NMR (125 MHz, CDCl_3_) δ 173.98 (CO-16), 173.87 (CO-1’), 72.31 (C-10’), 71.85 (C-7), 64.27 (C-1), 63.27 (C-16’), 37.48 (C-8), 37.06 (C-6), 34.33 (C-15), 28.56 (C-2), 37.34, 37.09, 32.81, 29.63, 29.58, 29.45, 29.22, 29.13, 29.19, 29.17, 29.07, 28.56, 25.96, 25.52, 25.30, 24.98 and 24.92 (C-3 to C-5; C-9 to C-14; C-2’ to C-9’ and C-11’ to C-15’), 60.13 (–OCH_2_CH_3_), 14.23 (–OCH_2_CH_3_), 25.96 SiC(Me)_3_-16’, 18.35 SiC(Me)_3_−16’, -5.28 Si(Me)_2_−16’; 25.91 SiC(Me)_3_−10’, 18.13 SiC(Me)_3_−10’ and −4.43 Si(Me)_2_−10’.

Deprotection of 16-ethoxy-7-hydroxy-16-oxohexadecyl 10’,16’-bis(tert-butyldimethylsilyloxy hexadecanoate) (**7a**). Compound **7a** (30 mg) was dissolved in 100 mL of CH_2_Cl_2_, and 0.5 mL of hydrofluoric acid was added. The reaction was stopped after 5 min, and product **7b** was extracted and purified.

*16-Ethoxy-7-hydroxy-16-oxohexadecyl-10’,16’-dihydroxyhexadecanoate* (**7b**). Colorless oil, (19 mg, 92%). ^1^H-NMR (500 MHz, CDCl_3_) δ 4.11 (q, *J* = 7.14 Hz, 2H) –CH_2_CH_3_, 4.05 (t, *J* = 6.72 Hz, 2H) CH_2_-1, 3.60 (t, *J* = 6.55 Hz, 4H) CH_2_-16’, CH-10’ and CH-7, 2.28 (t, *J* = 7.54 Hz, 4H) CH_2_-2’ and CH_2_-15, 1.61 (m, 4H), 1.50 (m, 4H), 1.46–1.27 (m, 44H), 1.26 (t, *J* = 7.14 Hz, 3H). ^13^C-NMR (125 MHz, CDCl_3_) δ 178.91 (CO-16), 173.53 (CO-1’), 72.30 (C-10’), 71.83 (C-7), 64.20 (C-1), 63.29 (C-16’), 60.09 (–OCH_2_CH_3_). HR-FABMS (calc. for C_34_H_66_O_7_: 586.4809), found 586.4809.

#### 3.3.5. Branched trimer of 10,16-DHPA derivatives

To a stirred solution of **3** (350 mg, 0.67 mmol) and **4** (106.3 mg, 0.33 mmol) in dry CH_2_Cl_2_ (4 mL), DMAP (82.1 mg, 0.67 mmol), and DCC (277.2 mg, 1.34 mmol) were added. The reaction mixture was then stirred at room temperature for five days, poured into NH_4_Cl saturated solution (5 mL), and extracted with dichloromethane (3 × 20 mL); the extract was washed with brine (2 × 10 mL), dried with anhydrous Na_2_SO_4_ after which the solvent was evaporated to dryness. The crude product was purified by radial chromatography (chromatotron), successively eluted with hexane, CH_2_Cl_2_, and CH_2_Cl_2_-MeOH (9:1 and 4:1; v:v).

*16-Ethoxy-16-oxohexadecane-1,7-diyl-bis(10’,16’-tert-butyldimethylsilyl-oxy)hexadecanoate* (**8a**). ^1^H-NMR (500 MHz, CDCl_3_) δ 4.85 (quint, *J* = 6.50 Hz, 1H) H-7, 4.11 (q, *J* = 7.14 Hz, 2H) –OCH_2_CH_3_, 4.04 (t, *J* = 6.77 Hz, 2H) CH_2_-1, 3.59 (m, 6H) CH_2_-16’, CH_2_-16’’, CH-10’ and CH-10’’, 2.28 (m, 6H) CH_2_-2’, CH_2_-2’’ and CH_2_-15, 1.61 (m, 12H) CH_2_-15’, CH_2_-15’’, CH_2_-11’, CH_2_-11’’, CH_2_-9’ and CH_2_-9’’, 1.50 (m, 6H) CH_2_-3’, CH_2_-3’’ and CH_2_-14, 1.46-1.27 (m, 57H), 0.89 (s, 18H) Si-tBu, 0.88 (s, 18H) Si-tBu, 0.04 (s, 12H) SiMe_2_ and 0.03 (s, 12H) SiMe_2_. ^13^C-NMR (125 MHz, CDCl_3_) Monomer CO_2_Et terminal δ 173.94 (CO-1’’), 173.84 (CO-16), 173.66 (CO-1’), 73.89 (C-7), 72.35 (C-10’ and C-10’’), 64.27 (C-2), 63.29 (C-16’), 60.13 (C-16’’), 34.71 (C-15), 34.37 (C-2’ and C-2’’), 37.14, 37.10, 32.92, 29.63, 29.85, 29.52, 29.49, 29.21, 29.19, 29.13, 29.11, 29.07, 28.59, 25.62, 25.33, 25.31, 25.11, and 24.94 (C-3 to C-5, C-9 to C-14, C-3’ to C-9’, C-11’ to C-15’, C-3’’ to C-9’’ and C-11’’ to C-15’’), 63.29 (–OCH_2_CH_3_), 14.26 (–OCH_2_CH_3_), 25.98 SiC(Me)_3_−16’, 18.37 SiC(Me)_3_−16’, −5.26 Si(Me)_2_-16’, 25.94 SiC(Me)_3_−10’, 18.15 SiC(Me)_3_−10’, −4.41 Si(Me)_2_−10’, 25.98 SiC(Me)_3_−16’’, 18.37 SiC(Me)_3_−16’’, −5.26 Si(Me)_2_−16’’, 25.94 SiC(Me)_3_−10’’, 18.16 SiC(Me)_3_−10’’ and -4.41 Si(Me)_2_−10’’.

Deprotection of compound **8a** (60 mg) was carried out following the protocol previously described for deprotection of compound **7a**.

*16-Ethoxy-16-oxohexadecane-1,7-diyl bis(10’,16’-dihydroxyhexadecanoate)* (**8b**). White wax (35 mg, 87.4%). ^1^H-NMR (500 MHz, CDCl_3_) δ 4.85 (quint, *J* = 6.50 Hz, 1H) CH-7, 4.12 (q, *J* = 7.14 Hz, 2H) –OCH_2_CH_3_, 4.04 (t, *J* = 6.77 Hz, 2H) CH_2_-1, 3.59 (m, 8H) CH_2_-16’, CH_2_-16’’, CH_2_-10’ and CH_2_-10’, 2.27 (m, 6H) CH_2_-2’, CH_2_-2’ ’ and CH_2_-15, 1.61 (m, 12H) CH_2_-15’, CH_2_-15’’, CH_2_-11’, CH_2_-11’’, CH_2_-9’ and CH_2_-9’’, 1.50 (m, 6H) CH_2_-3’, CH_2_-3’’ and CH_2_-14, 1.46-1.27 (m, 57H). ^13^C-NMR (125 MHz, CDCl_3_) δ 173.94 (CO-1’’), 173.85 (CO-16), 173.66 (CO-1’), 73.89 (C-7), 72.35 (C-10’ and C-10’’), 64.27 (C-2), 63.33 (C-16’ and C-16’’), 34.71 (C-15), 34.37 (C-2’ and C-2’’), 37.14, 37.10, 32.92, 29.63, 29.85, 29.52, 29.49, 29.21, 29.19, 29.11, 29.13, 29.11, 29.07, 28.59, 25.62, 25.31, 25.33, 25.11, 24.95 and 24.94 (C-3 to C-5, C-9 to C-14, C-3’ to C-9’, C-11’ to C-15’, C-3’’ to C-9’’ and C-11’’ to C-15’’), 63.30 (–OCH_2_CH_3_) and 14.26 (–OCH_2_CH_3_). HR-FABMS (calc. for C_50_H_96_O_10_: 856.7003), found 856.7002.

#### 3.3.6. Linear trimer of 10,16-DHPA derivatives

To a stirred solution of **7b** (50 mg, 0.08 mmol) and **3** (14.8 mg, 0.08 mmol) in dry CH_2_Cl_2_ (4 mL), DMAP (5.7 mg, 0.04 mmol) and DCC (17.5 mg, 0.08 mmol) were added. The reaction mixture was then stirred for six days at room temperature. The reaction was stopped, poured into an NH_4_Cl solution (5 mL), and extracted with dichloromethane (3 × 20 mL) after which the extract was washed with water (2 × 10 mL), dried over anhydrous Na_2_SO_4_ and evaporated *in vacuo*. The crude product was purified by radial chromatography (chromatotron) through successive elutions with hexane, CH_2_Cl_2_, and CH_2_Cl_2_-MeOH (9:1 and 4:1; v:v).

*16-Ethoxy-7-hydroxy-16-oxohexadecyl 16-((10,16-bis((tert-butyldimethylsilyl)oxy)hexadecanoyl) oxy)-10-hydroxyhexadecanoate* (**10**). Colorless oil (9.2 mg, 10%)^ 1^H-NMR (500 MHz, CDCl_3_) monomer CO_2_Et terminal: δ ppm 4.11 (q, *J* = 7.14 Hz, 2H) –OCH_2_CH_3_, 4.05 (t, *J* = 6.72 Hz, 2H) CH_2_-1, 3.60 (t, *J* = 6.55 Hz, 5H) CH_2_-16’’, CH-10’’, CH-10’ and CH-7, 2.28 (t, *J* = 7.54 Hz, 6H) CH_2_-2’’, CH_2_-2’ and CH_2_-15, 1.61 (m, 4H) CH_2_-15’ and CH_2_-2, 1.50 (m, 4H) CH_2_-3’ and CH_2_-14, 1.46-1.27 (m, 44H), 1.26 (t, *J* = 7.14 Hz, 3H) –OCH_2_CH_3_, 0.89 (s, 9H) Si-tBu, 0.88 (s, 9H) Si-tBu, 0.05 (s, 6H) SiMe_2_ and 0.03 (s, 6H) SiMe_2_. ^13^C-NMR (125 MHz, CDCl_3_) δ 174.01 (CO-16), 173.97 (CO-1’), 173.87 (CO-1’), 72.34 (C-10’), 72.31 (C-10’), 71.80 (C-7), 64.25 (C-1), 63.27 (C-16’), 63.25 (C-16’), 37.45 (C-8), 37.06 (C-6), 34.33 (C-15), 28.56 (C-2), 37.34, 37.09, 32.81, 29.63, 29.58, 29.45, 29.22, 29.17, 29.13, 29.19, 29.07, 28.56, 25.96, 25.79, 25.52, 25.30, 24.98 and 24.92 (C-3 to C-5, C-9 to C-14, C-2’ to C-9’, C-11’ to C-15’, C-2’’ to C-9’’ and C-11’’ to C-15’’), 60.13 (–OCH_2_CH_3_), 14.23 (–OCH_2_CH_3_), 25.97 SiC(Me)_3_−16’’, 18.38 SiC(Me)_3_-16’’, −5.26 Si(Me)_2_−16’’; 25.93 SiC(Me)_3_−10’’, 18.15 SiC(Me)_3_−10’’ and −4.42 Si(Me)_2_−10’’.

## 4. Conclusions

Here, we have shown a simple, efficient protocol to perform controlled dimerization and trimerization, as well as the selective oxidation of 10,16-DHPA.

The above simple reactions took advantage of the hydroxyl groups at the C-10 and C-16 positions which were strategically functionalized in order to produce the monomers 7-oxohexadecanedioic and 10-oxo-16-hydroxyhexadecanoic acids, as well as a dimer and branched and linear trimers, which were fully characterized by different spectroscopic analysis. 10-Oxo-16-hydroxyhexadecanoic acid, the dimer and linear trimer could be useful in the preparation of aliphatic polyesters while the branched trimer can lead to the formation of dendrimers, and the 7-oxohexadecanedioic would be useful to obtain linear polyester with some diols. The effect of the products obtained here in will give to the polyesters functionalizable keto or hydroxyl moieties which can be modified, derivatized, or cross-linked at these sites, to produce polymers with bioactive moieties for medical applications.

## Supplementary Materials

Supplementary Materials can be found at http://www.mdpi.com/1420-3049/16/6/4923/s1.

## References

[1] Narayan N. (2001). Drivers for biodegradable/compostable plastics and role of composting in waste management and sustainable agriculture. Orbit J..

[2] Gross R.A., Kalra B. (2002). Biodegradable polymers for the environment. Science.

[3] van der Walle G.A., de Koning G.J., Weusthuis R.A., Eggink G. (2002). Properties, modifications and applications of biopolyesters. Adv. Biochem. Eng. Biotechnol..

[4] Bordes P., Pollet E., Averous L. (2009). Nano-biocomposites: Biodegradable polyester/nanoclay systems. Prog.Polymer Sci..

[5] Mahapatro A., Kalra B., Kumar A., Gross R.A. (2003). Lipase-catalyzed polycondensations: Effect of substrates and solvent on chain formation, dispersity, and end-group structure. Biomacromolecules.

[6] Moon S.I., Lee C.W., Taniguchi I., Miyamoto M., Kimura Y. (2001). Melt/Solid polycondensation of L-lactic acid: An alternative route to poly(L-lactic acid) with high molecular weight. Polymer.

[7] Yang Y., Lu W., Zhang X., Xie W., Cai M., Gross R.A. (2010). Two-step biocatalytic route to biobased functional polyesters from ω-carboxy fatty acids and diols. Biomacromolecules.

[8] Uyama H., Takeya S., Kobayashi S. (1993). Synthesis of polyesters by enzymic ring-opening copolymerization using lipase catalyst. Proc. Jpn. Acad. Ser. B Phys. Biol. Sci..

[9] Rehm B.H. (2003). Polyester synthases: Natural catalysts for plastics. Biochem.J..

[10] Bernards M.A., Lewis N.G. (1998). The macromolecular aromatic domain in suberized tissue: A changing paradigm. Phytochemistry.

[11] Gerard H.C., Osman S.F., Fett W.F., Moreau R.A. (1992). Separation, identification and quantification of monomers from cutin polymers by high performance liquid chromatography and evaporative light scattering detection. Phytochem.Anal..

[12] Kolattukudy P.E. (2001). Polyesters in higher plants. Adv. Biochem. Eng Biotechnol..

[13] Fang X., Qiu F., Yan B., Wang H., Mort A.J., Stark R.E. (2001). NMR studies of molecular structure in fruit cuticle polyesters. Phytochemistry.

[14] Kallio H., Nieminen R., Tuomasjukka S., Hakala M. (2006). Cutin composition of five finnish berries. J. Agric. Food Chem..

[15] Osman S.F., Irwin P., Fett W.F., O’Connor J.V., Parris N. (1999). Preparation, isolation, and characterization of cutin monomers and oligomers from tomato peels. J. Agric. Food Chem..

[16] Ray A.K., Stark R.E. (1998). Isolation and molecular structure of an oligomer produced enzymatically from the cuticle of lime fruit. Phytochemistry.

[17] Suh M.C., Samuels A.L., Jetter R., Kunst L., Pollard M., Ohlrogge J., Beisson F. (2005). Cuticular lipid composition, surface structure, and gene expression in Arabidopsis stem epidermis. Plant Phys..

[18] Mahapatro A., Kumar A., Gross R.A. (2004). Mild, solvent-free omega-hydroxy acid polycondensations catalyzed by candida antarctica lipase B. Biomacromolecules.

[19] Benitez J.J., Heredia-Guerrero J.A., Serrano F.M., Heredia A. (2008). The role of hydroxyl groups in the self-assembly of long chain alkylhydroxyl carboxylic acids on Mica. J. Phys. Chem. C.

[20] Heredia-Guerrero J.A., Heredia A., García-Segura R., Benitez J.J. (2009). Synthesis and characterization of a plant cutin mimetic polymer. Polymer.

[21] Douliez J.P. (2004). Cutin and suberin monomers are membrane perturbants. J. Colloid Interface Sci..

[22] Osman S.F., Gerard H.C., Fett W.F., Moreau R.A., Dudley R.L. (2002). Method for the production and characterization of tomato cutin oligomers. J. Agric. Food Chem..

[23] Stark R.E., Yan B., Ray A.K., Chen Z., Fang X., Garbow J.R. (2000). NMR studies of structure and dynamics in fruit cuticle polyesters. Solid State Nucl.Magn. Reson..

[24] Croteau R., Kolattukudy P.E. (1974). Biosynthesis of hydroxyfatty acid polymers. Enzymatic synthesis of cutin from monomer acids by cell-free preparations from the epidermis of Vicia faba leaves. Biochemistry.

[25] Tian S., Fang X., Wang W., Yu B., Cheng X., Qiu F., Mort A.J., Stark R.E. (2008). Isolation and identification of oligomers from partial degradation of lime fruit cutin. J. Agric. Food Chem..

[26] Matic M. (1956). The chemistry of plant cuticles: A study of cutin from *Agave americana* L. Biochem. J..

[27] Ahmed A., Crawford T., Gould S., Ha Y.S., Hollrah M., Noor-E-Ain., Dickman M.B., Dussault P.H. (2003). Synthesis of (*R*)- and (*S*)-10,16-dihydroxyhexadecanoic acid: Cutin stereochemistry and fungal activation. Phytochemistry.

[28] Benitez J.J., Garcia-Segura R., Heredia A. (2004). Plant biopolyester cutin: A tough way to its chemical synthesis. Biochim.Biophys. Acta.

[29] Benitez J.J., Heredia-Guerrero J.A., Heredia A. (2007). Self-Assembly of carboxylic acids and hydroxyl derivatives on Mica. A Qualitative AFM Study. J. Phys. Chem. C.

[30] Deshmuk A.P., Simpson A.J., Hatcher P.G. (2003). Evidence for cross-linking in tomato cutin using HR-MAS NMR spectroscopy. Phytochemistry.

[31] Graca J., Lamosa P. (2010). Linear and branched poly(w-hydroxyacid) ester in plant cutins. J. Agric. Food Chem..

