# Supplementary figures and images for: Derivatives of 10,16-Dihydroxyhexadecanoic Acid Isolated from Tomato (Solanum lycopersicum) as Potential Material for Aliphatic Polyesters
Source: Molecules. 2011 Jun 15;16(6):4923–36. doi: 10.3390/molecules16064923 (PMC6264701; doi:10.3390/molecules16064923)

## Supplementary Materials

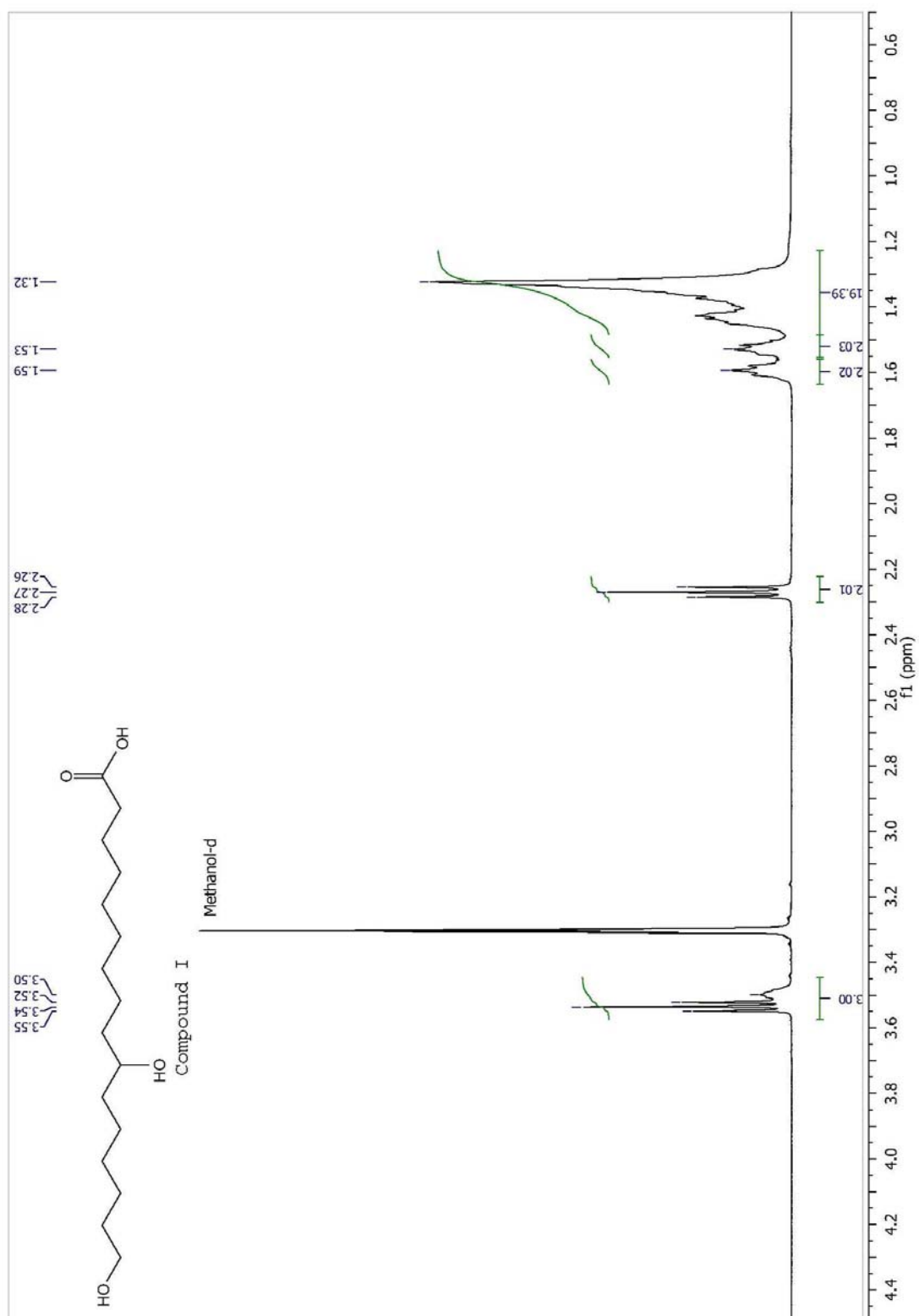

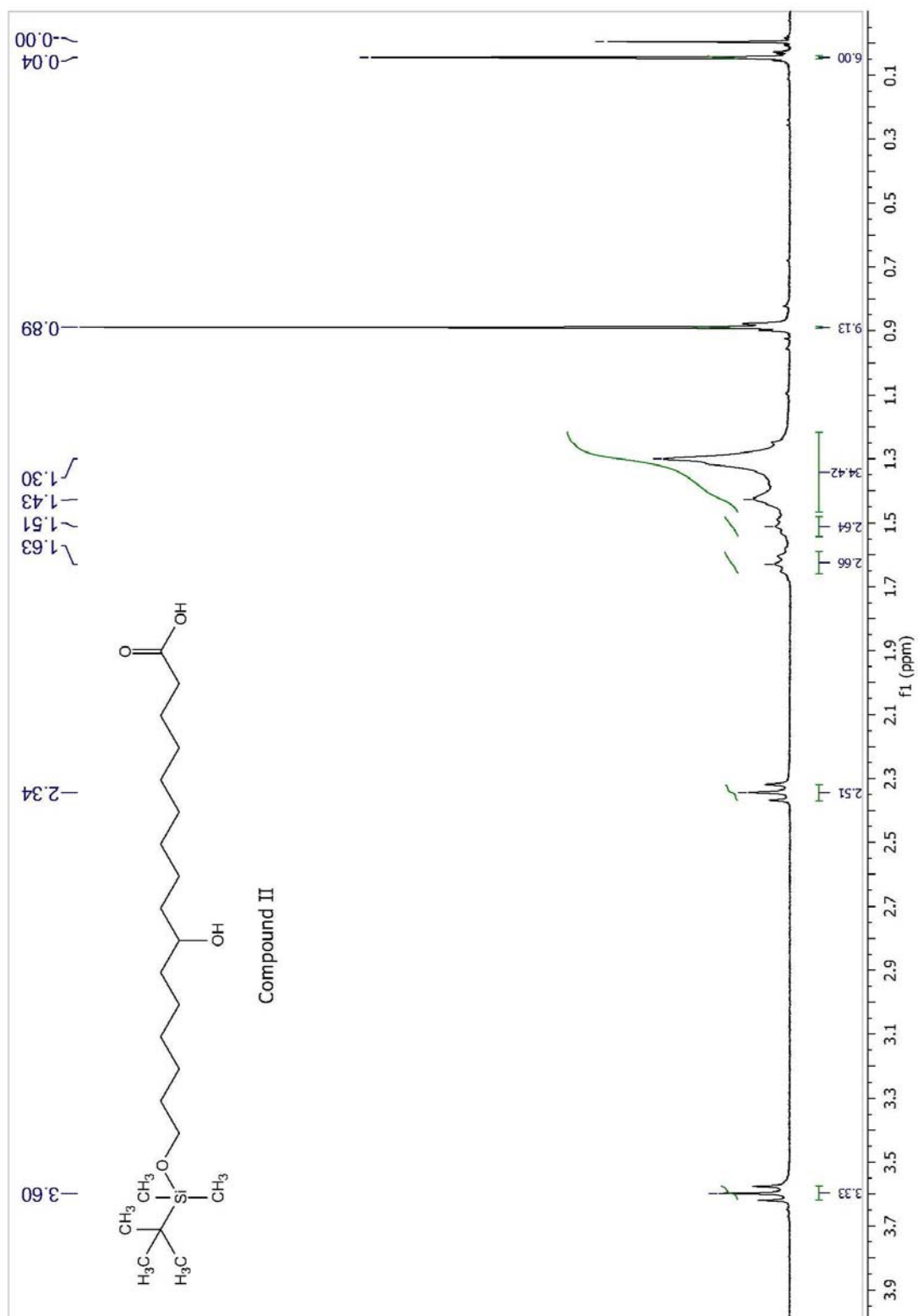

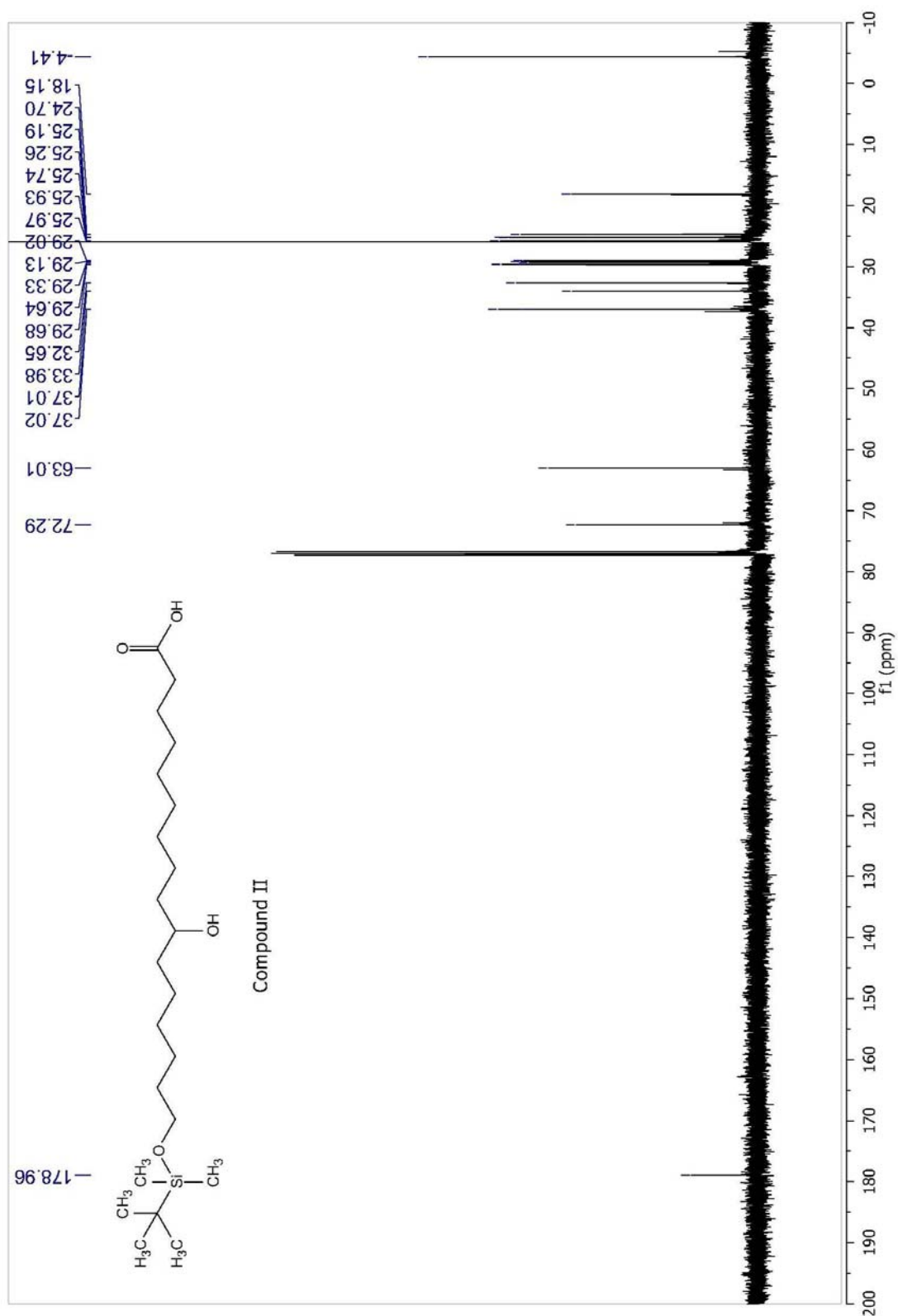

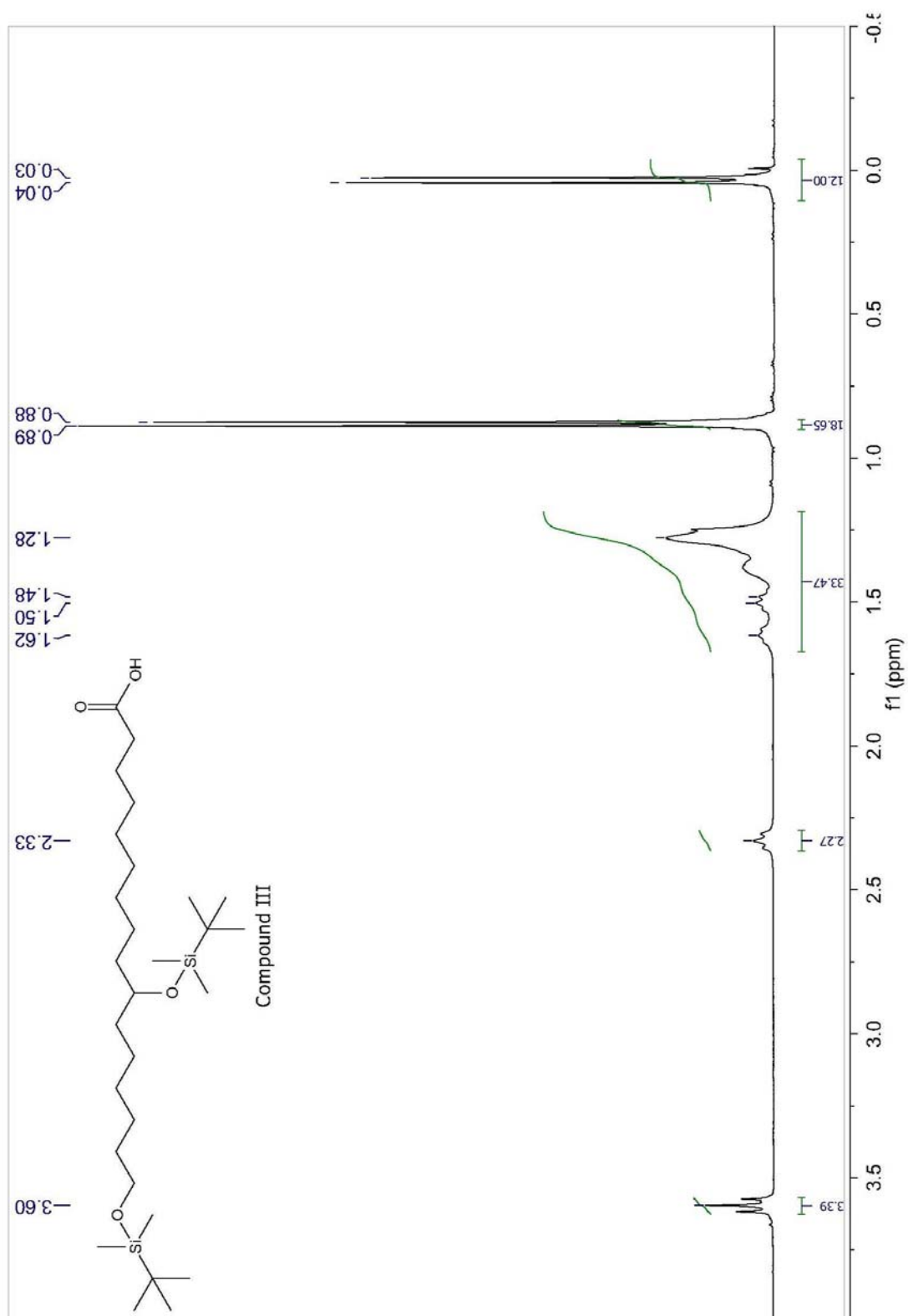

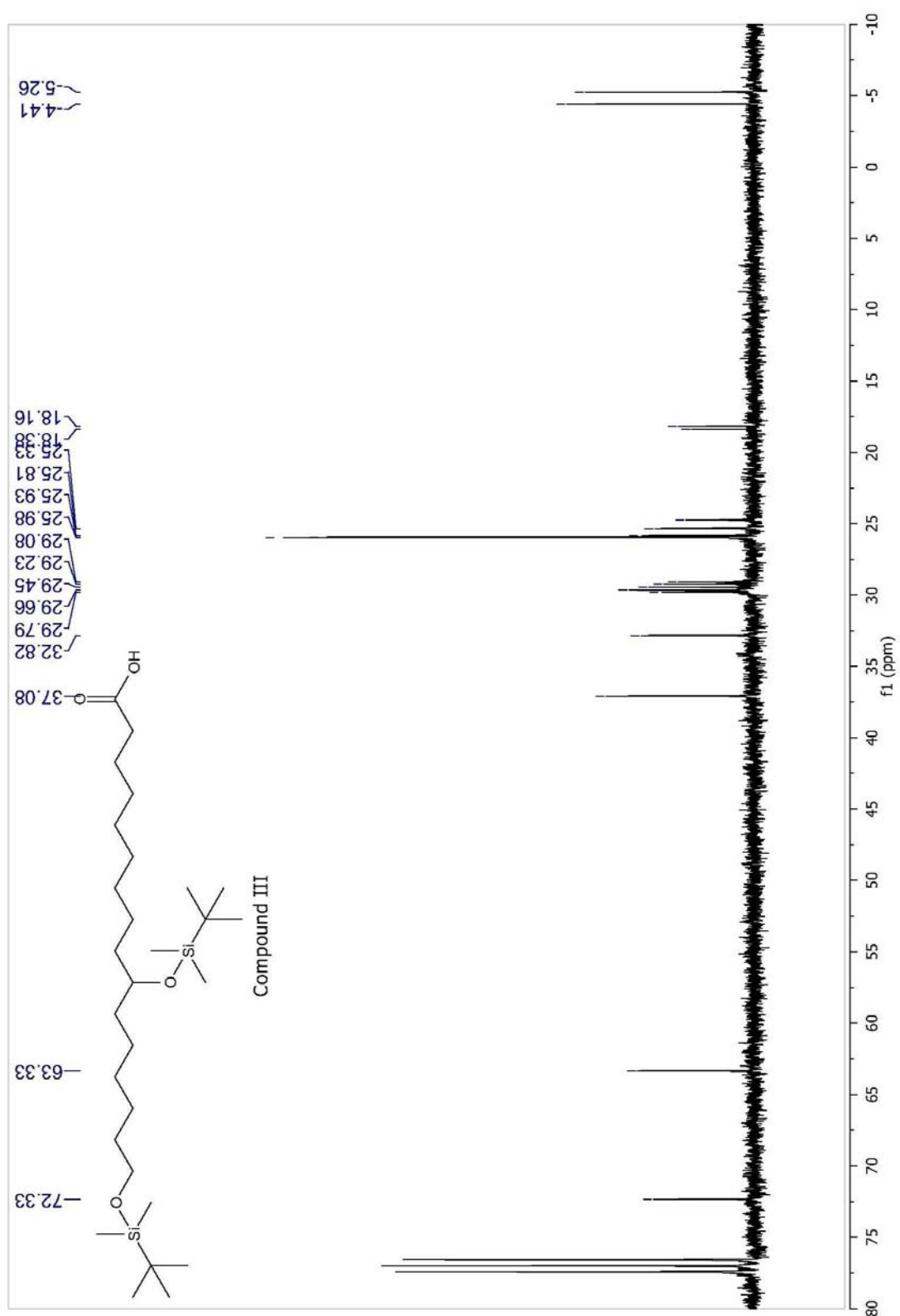

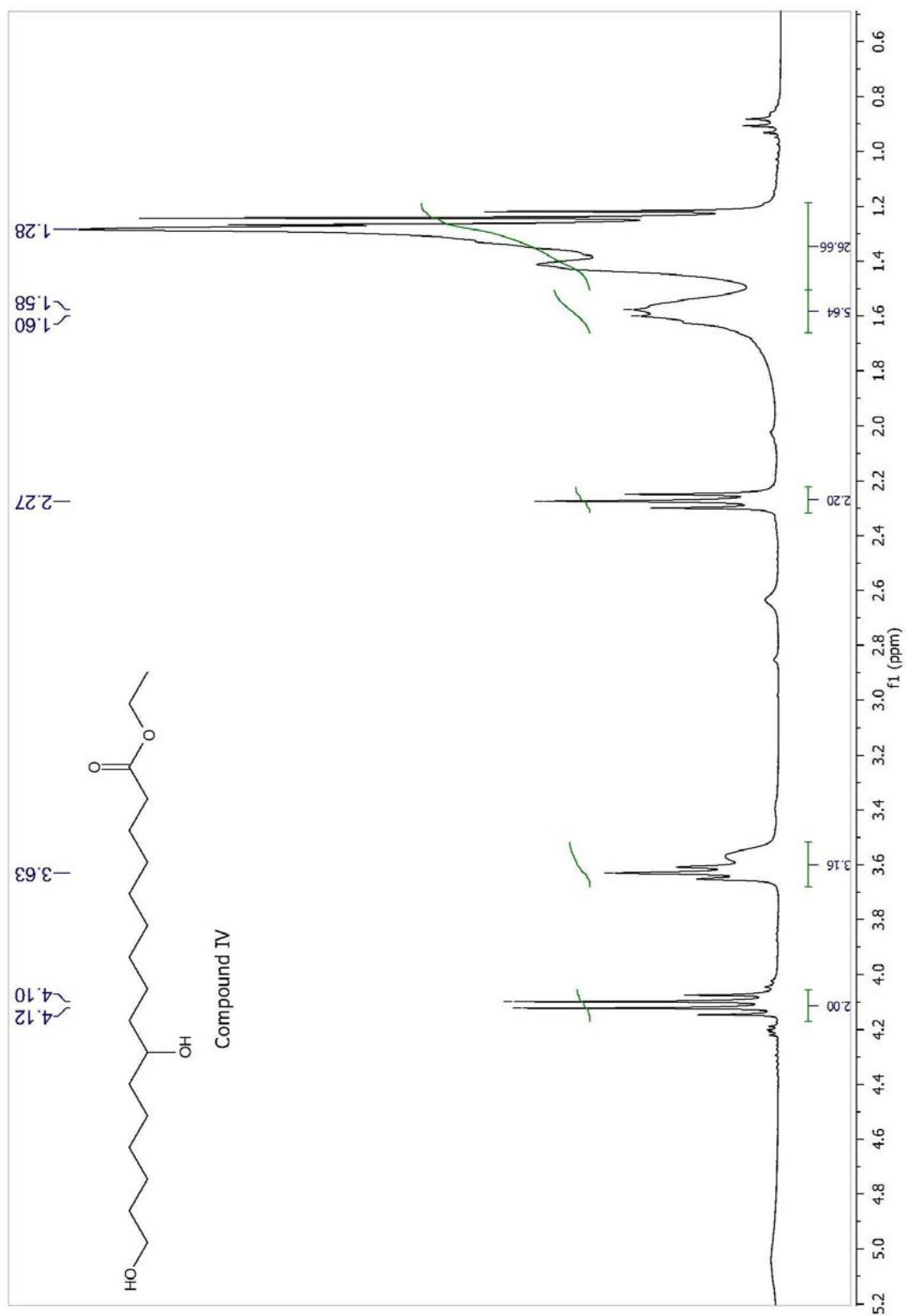

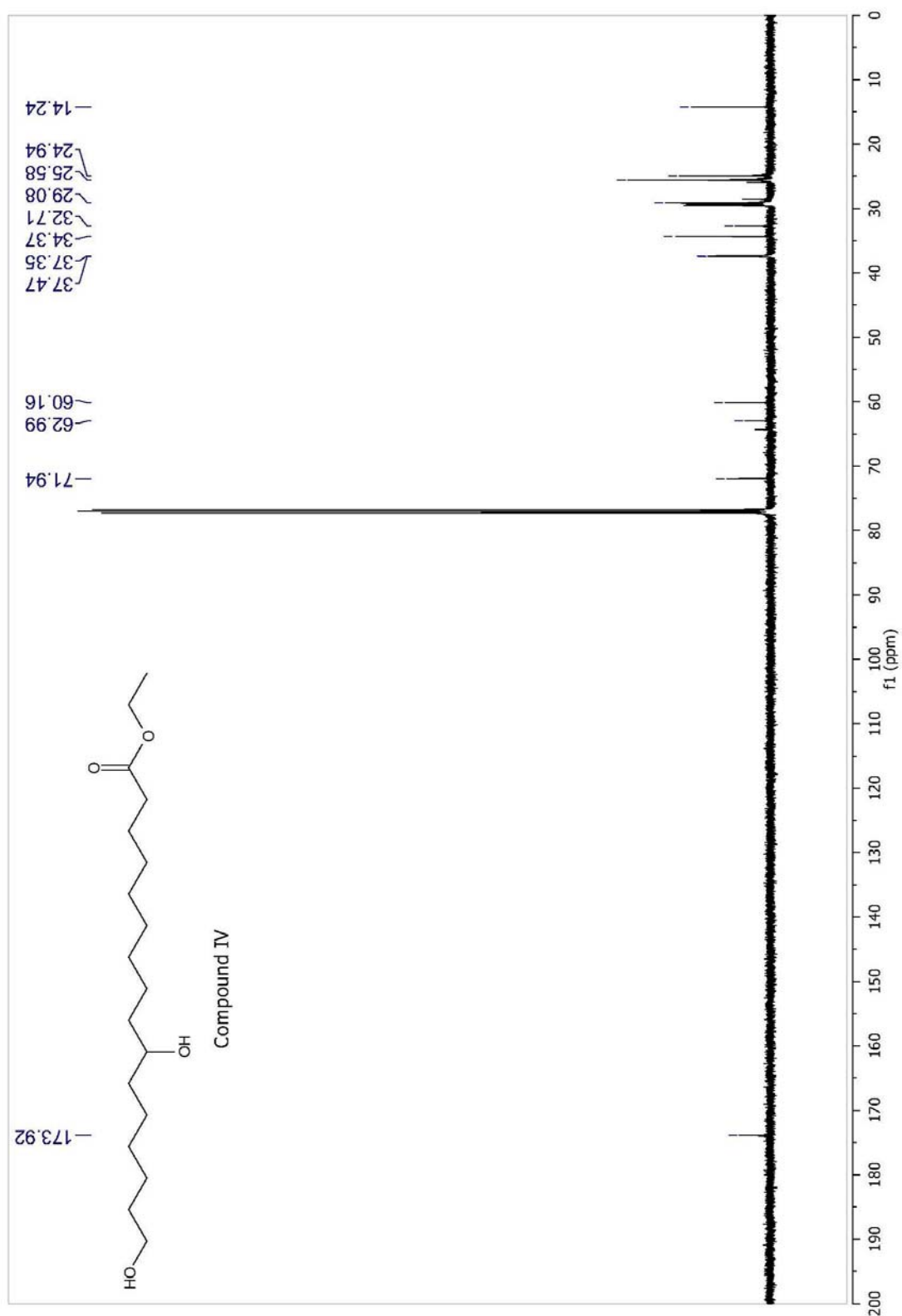

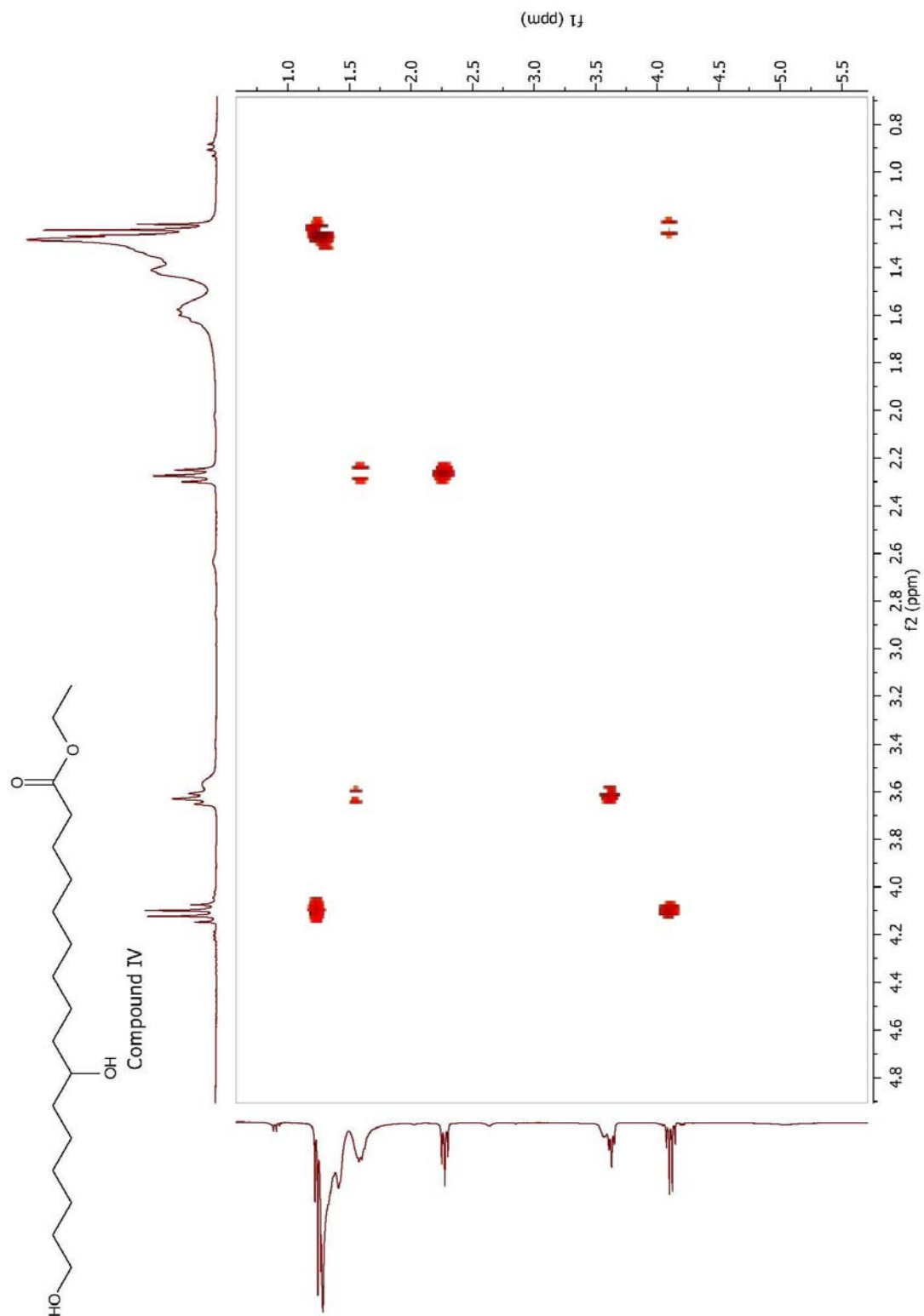

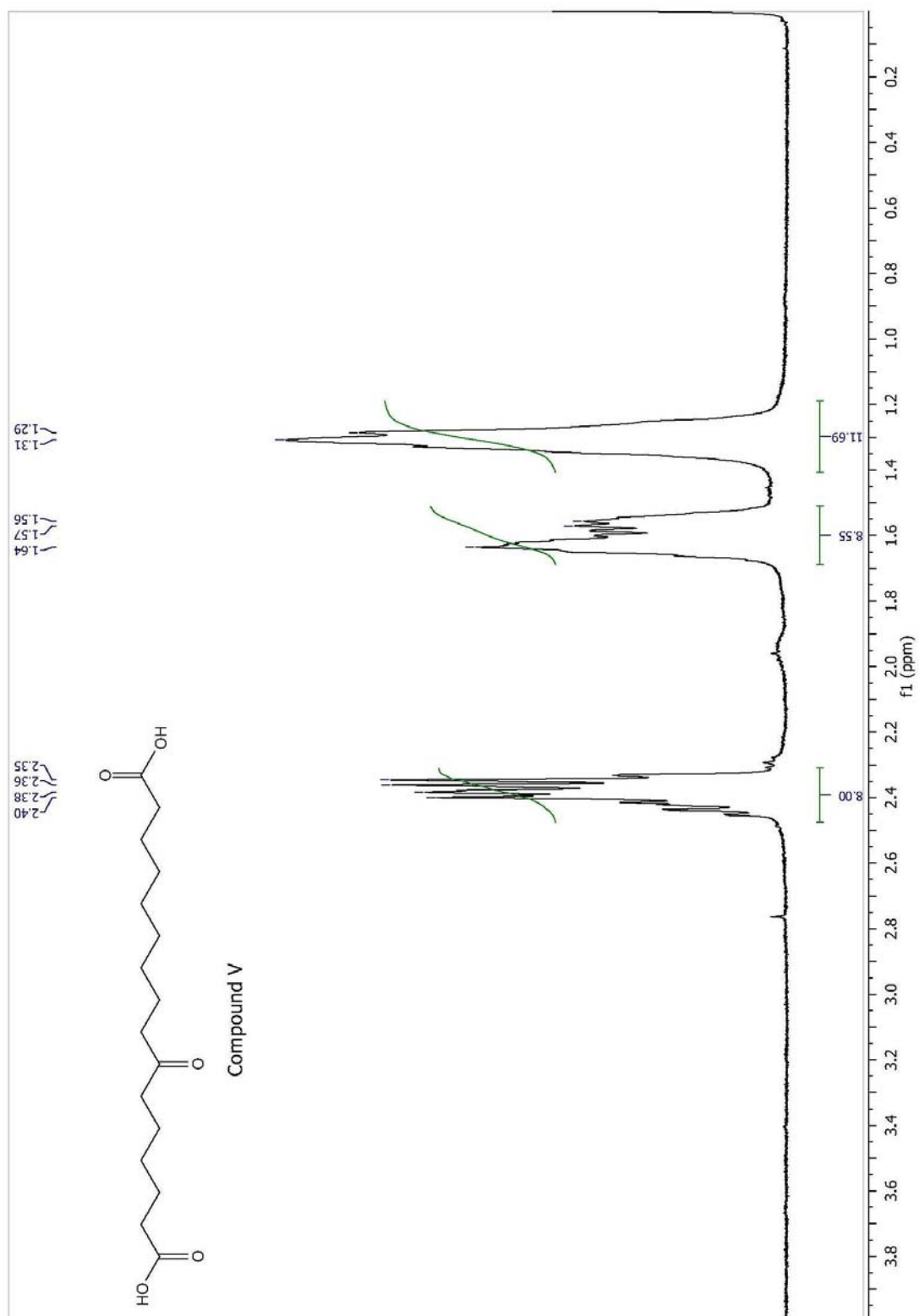

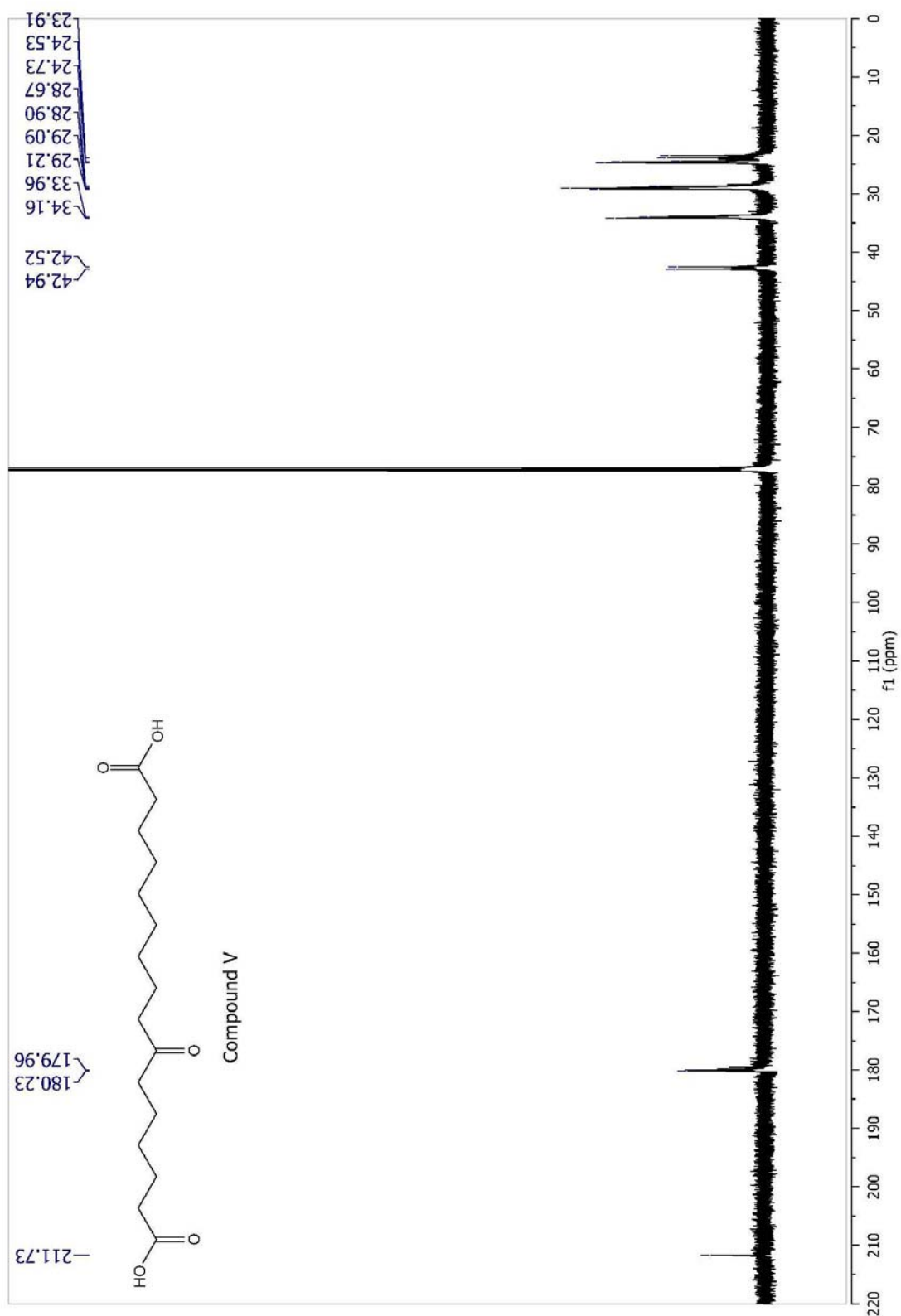

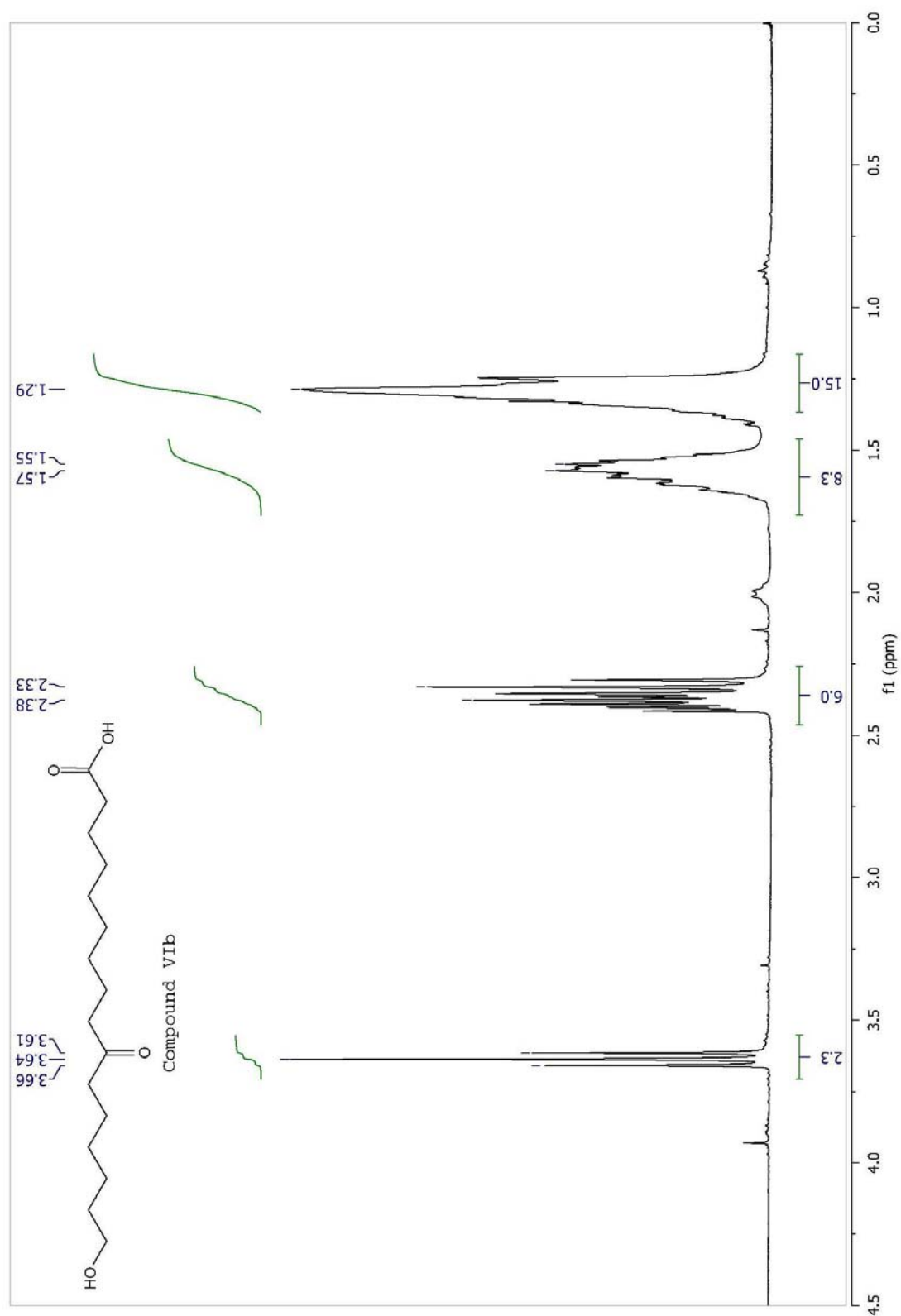

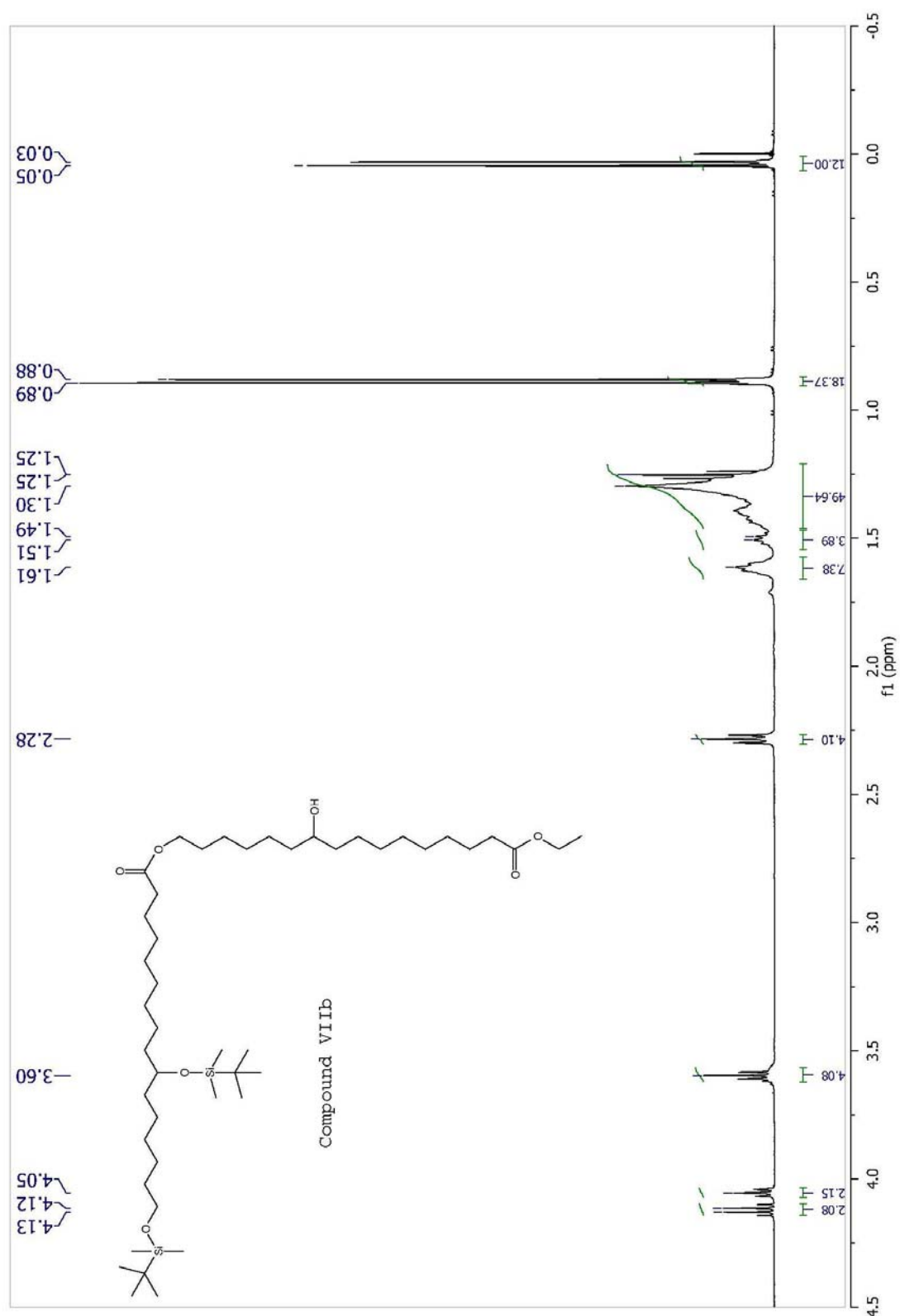

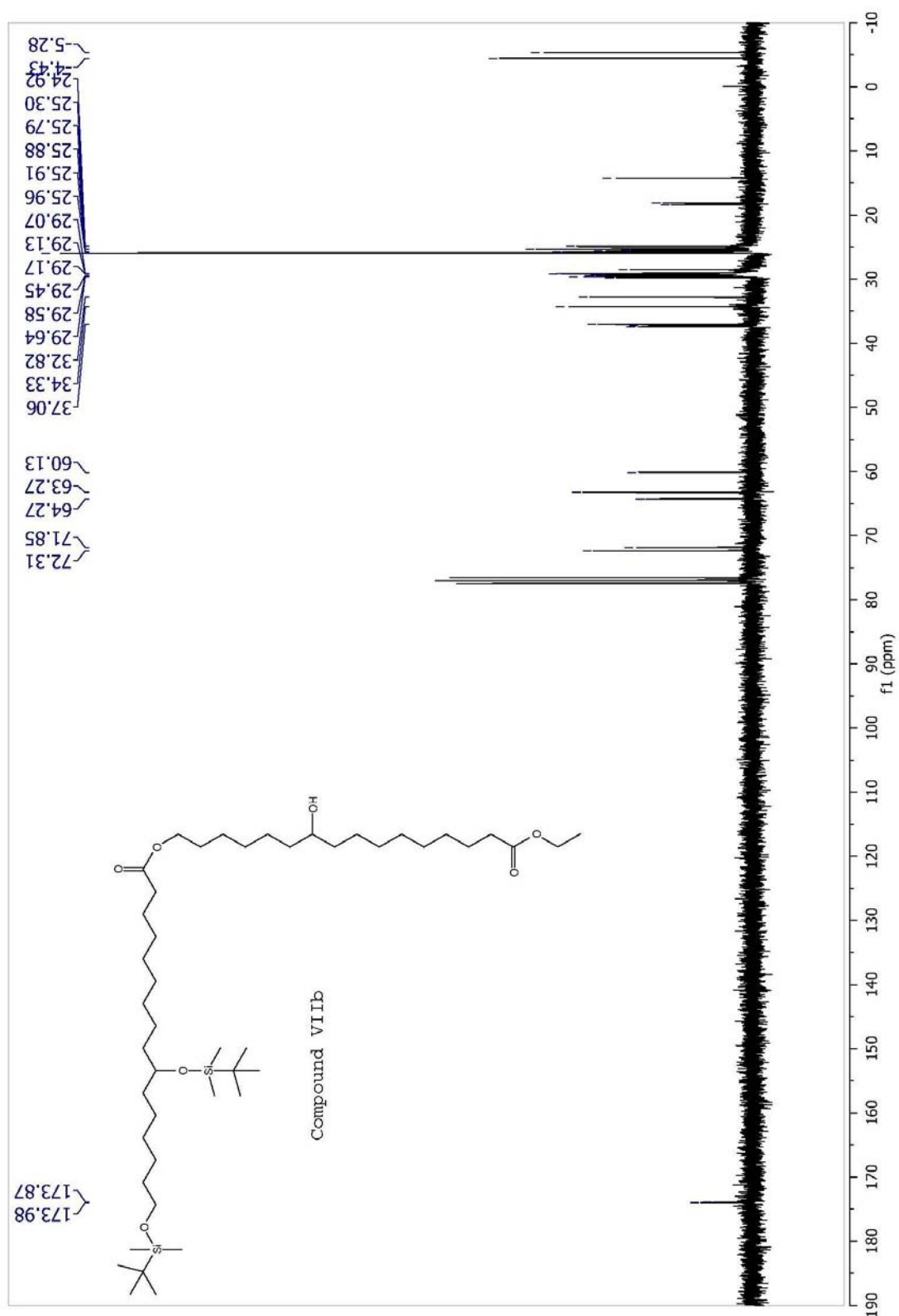

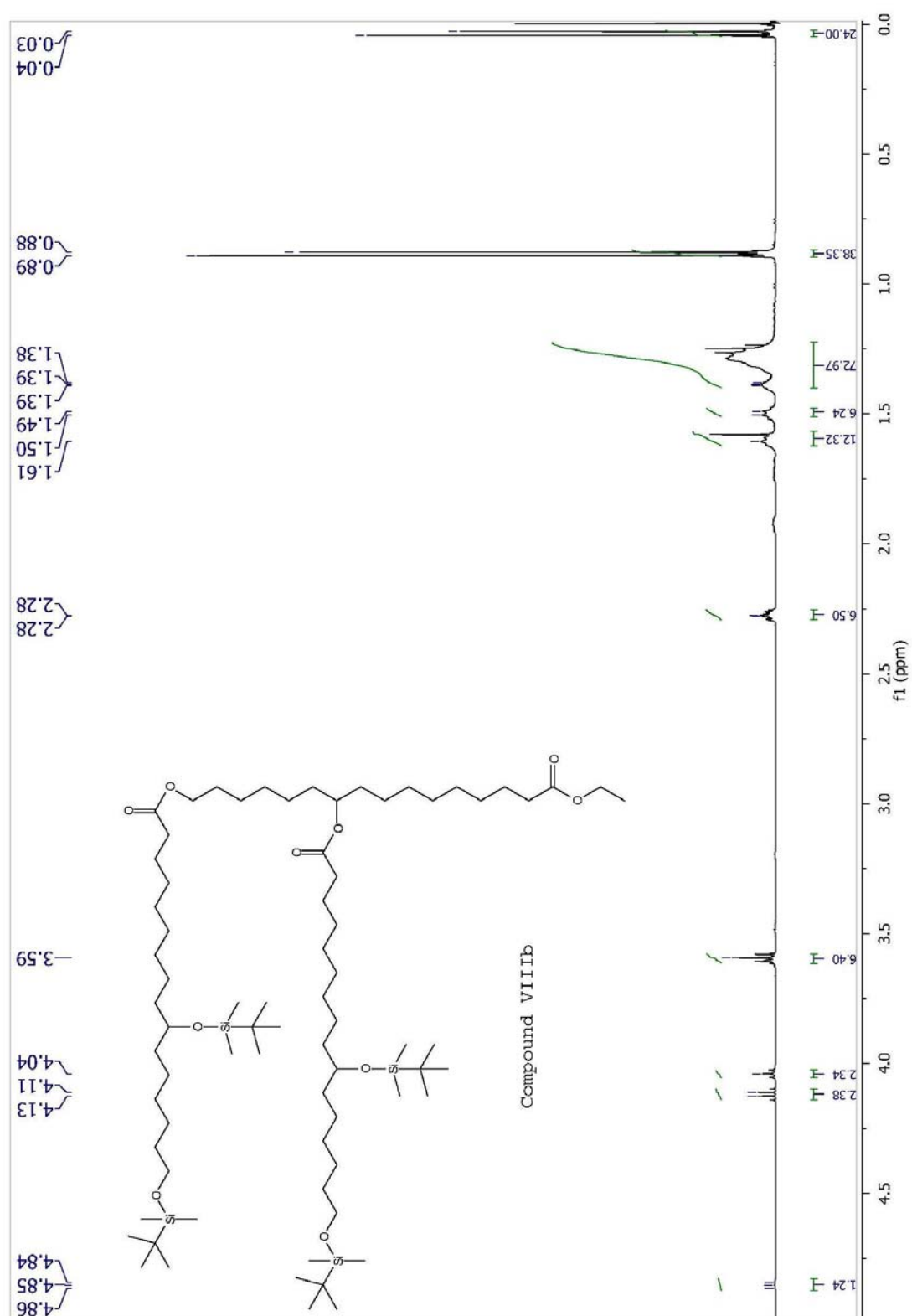

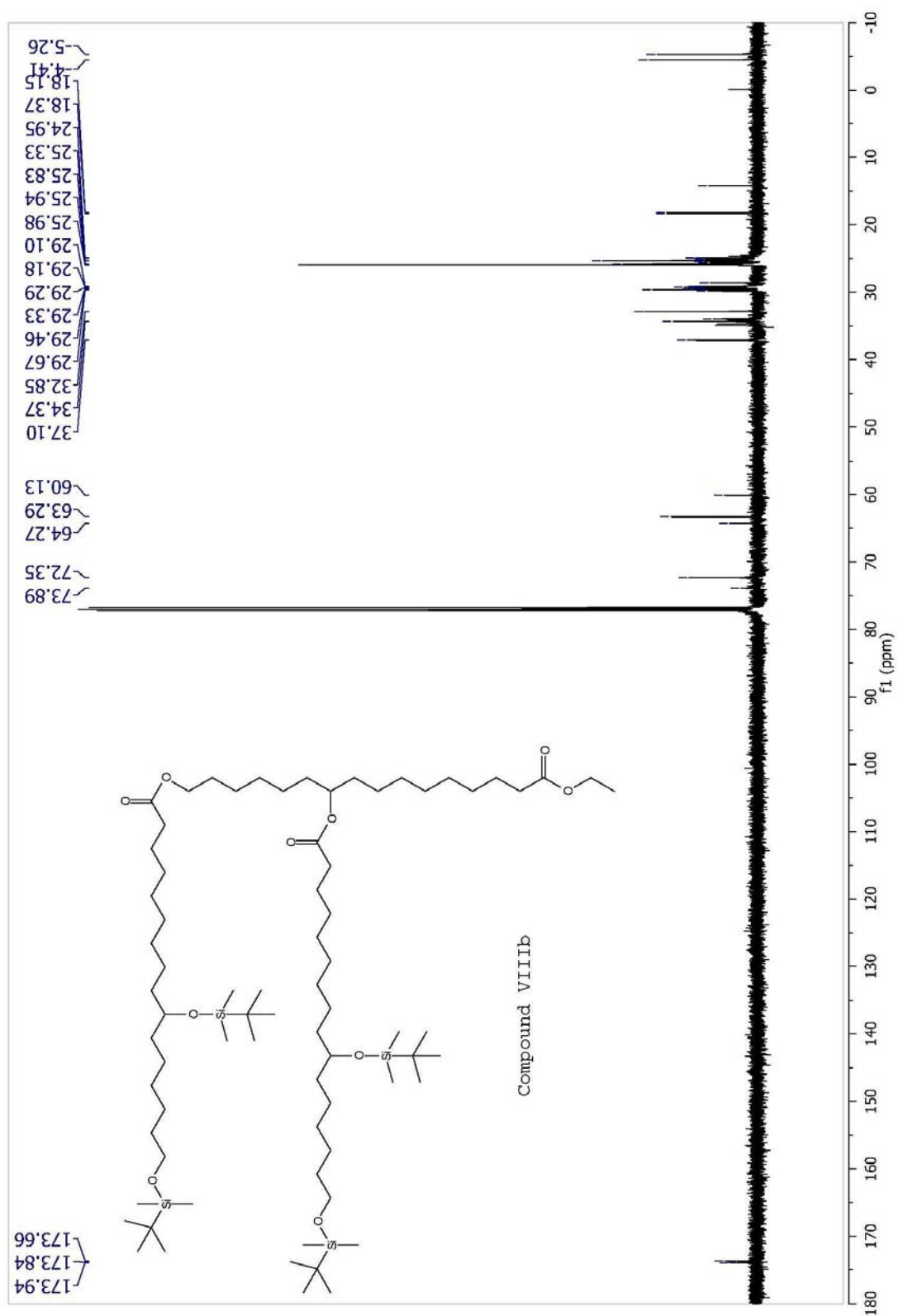

Supplement: Supplementary file 1 [file molecules-16-04923-s001.pdf]
